# IR783‐Stabilized Nanodrugs Enhance Anticancer Immune Response by Synergizing Oxidation Therapy and Epigenetic Modulation

**DOI:** 10.1002/advs.202415684

**Published:** 2025-03-27

**Authors:** Jinzhao Liu, Meicen Wu, Qingyang Lyu, Chang Yang, Ni Fan, Kang Chen, Weiping Wang

**Affiliations:** ^1^ State Key Laboratory of Pharmaceutical Biotechnology The University of Hong Kong Hong Kong China; ^2^ Department of Pharmacology and Pharmacy Li Ka Shing Faculty of Medicine The University of Hong Kong Hong Kong China; ^3^ Dr. Li Dak‐Sum Research Centre The University of Hong Kong Hong Kong China; ^4^ Department of Medicine Li Ka Shing Faculty of Medicine The University of Hong Kong Hong Kong China

**Keywords:** cancer immunotherapy, epigenetic regulation, immunogenic cell death, oxidation therapy, small molecule self‐assembly, synergistic effect

## Abstract

Immune evasion and metastasis are the leading causes of poor prognosis in triple‐negative breast cancer treatment. Since current standard immunotherapies have limited efficacy due to immunologically cold microenvironment, it is crucial to explore new strategies to sensitize anticancer immune response. In this study, it is found that incorporating *β*‐lapachone‐based oxidation therapy with CUDC101‐initiated epigenetic regulation results in synergistic antitumor effects and potent immune activation. To co‐deliver these two hydrophobic drugs, IR783 with cyanine structure serves as the stabilizer to form a nanoformulation based on small molecule self‐assembly. Such IR783‐stabilized nanodrugs can not only lead to cancer cell apoptosis through HDAC inhibition‐enhanced oxidation therapy but also cooperatively induce immunogenic cell death and promote pro‐inflammatory cytokine gene expression to reshape immunosuppressive microenvironment. Besides, nanodrugs can inhibit both primary and distant tumor growth effectively by elevating systemic anticancer immunity. This study provides a promising approach to synergize oxidation therapy with epigenetic modulation for safe and efficient breast cancer immunotherapy.

## Graphical abstract

IR783‐stabilized nanodrugs composed of NIR dye IR783, ROS inducer *β*‐lapachone, and epigenetic modulator CUDC101 are designed for breast cancer immunotherapy. The nanodrugs can not only promote cancer cell apoptosis through HDAC inhibition‐enhanced oxidation therapy but also reshape the immunosuppressive microenvironment, which provides a novel strategy to synergize oxidation therapy with epigenetic modulation for safe and efficient cancer therapy.

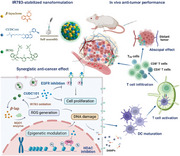

## 1 Introduction

Triple‐negative breast cancer (TNBC) is among the most aggressive tumors, characterized by high heterogeneity, multiple resistance, and fast invasion.^[^ ^1^ ^]^ Immune evasion and metastasis are the primary factors contributing to poor prognosis and treatment failure in TNBC patients.^[^ ^2^ ^]^ Therefore, cancer immunotherapy has been considered an effective approach to overcome the immunosuppressive microenvironment and has been widely used in clinics over the past decade. This includes treatments such as anti‐programmed death protein‐1/ligand‐1 (aPD‐1/PD‐L1) therapy and chimeric antigen receptor T (CAR‐T) cell therapy.^[^ ^3^ ^]^ However, only 20–30% of cancer patients respond favorably to aPD‐1/PD‐L1 therapies, primarily due to the scarcity of tumor antigen presentation and limited infiltrating cytotoxic T cells.^[^ ^4^ ^]^ Moreover, CAR‐T cell treatment has shown limited success in managing solid tumors, particularly in TNBC patients.^[^ ^5^ ^]^ Consequently, it is necessary to develop new strategies to enhance anticancer immune response. To achieve efficient cancer immunotherapy, enough tumor antigens are considerably needed to promote antigen presentation and subsequent T cell activation.^[^ ^6^ ^]^ Recent studies suggest that some chemotherapeutic drugs can help generate sufficient tumor antigens released from dying cancer cells, which is characterized as immunogenic cell death (ICD).^[^ ^7^ ^]^ One of the potential ICD inducers is *β*‐lapachone (*β*‐lap), which can produce reactive oxygen species (ROS) with the help of NAD(P)H: quinone oxidoreductase‐1 (NQO1) specifically in tumor tissues.^[^ ^8^ ^]^ Such oxidative stress can further initiate the release of damage‐associated molecular patterns (DAMPs) such as high‐mobility box1 (HMGB1) into the tumor microenvironment to activate antigen‐presenting cells (APCs) and boost systemic anticancer immunity.^[^ ^9^ ^]^

Apart from antigen presentation, the other consideration is preventing cancer cell metastasis. TNBC cells tend to be more aggressive at late stages and may spread to other parts of the body, such as the lungs, bones, lymph nodes, and even the brain.^[^ ^10^ ^]^ The epidermal growth factor receptor (EGFR) signaling pathway is considered a powerful target for inhibiting metastasis. Both anti‐EGFR monoclonal antibodies (like cetuximab and panitumumab) and next‐generation EGFR tyrosine kinase inhibitors (TKIs) have shown promising therapeutic effects in metastasis inhibition in preclinical and clinical studies.^[^ ^11^ ^]^ Therefore, in this study, we tried to combine the ICD inducer *β*‐lap with an EGFR inhibitor CUDC101 to enhance antigen exposure and suppress tumor cell invasion for effective breast cancer treatment. In addition to EGFR inhibition, CUDC101 designed with a hydroxamic acid structure can also act as an inhibitor of histone deacetylases (HDACs).^[^ ^12^ ^]^ HDACs play a crucial role in epigenetic regulation and can influence cancer cell gene expression by relaxing chromatin structure without changing the DNA sequence.^[^ ^13^ ^]^ Some HDAC inhibitors have been developed and approved for the treatment of certain types of cancer by modulating the transcription of downstream oncogenes.^[^ ^14^ ^]^ Considering the potential relationship between oxidative stress and epigenetic modulation, we hypothesized that CUDC101‐mediated HDAC inhibition might amplify the DNA damage and cell apoptosis in the *β*‐lap‐provoked oxidation therapy, thus leading to synergistic antitumor effect and strong immune activation.

**Figure 1:**
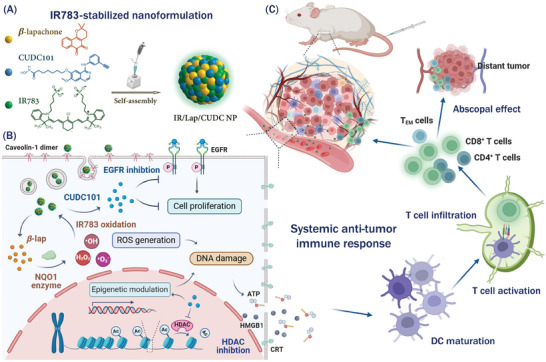
Schematic illustration of IR783‐stabilized nanodrugs to enhance anticancer immune response and promote breast cancer immunotherapy. A) The nanodrugs are formulated with IR783, *β*‐lap, and CUDC101. IR783 serves as a stabilizer to form nanoparticles with two hydrophobic drug molecules based on small molecule self‐assembly. B) The nanodrugs can enter breast cancer cells through CAV‐1‐mediated endocytosis pathway and achieve synergistic anticancer effects from NQO1‐dependent oxidation therapy and CUDC101‐provoked epigenetic regulation. C) The nanodrugs can further promote DAMPs release and dendritic cell maturation to boost systemic antitumor immunity and inhibit both primary and distant tumor growth.

Both *β*‐lap and CUDC101 are highly hydrophobic with low solubility and poor pharmacokinetics following systemic administration, which may considerably limit their applications for breast cancer treatment.^[^ ^15^ ^]^ To address this issue, we designed an IR783‐stabilized nanodrug formulation (denoted as IR/Lap/CUDC NPs) to co‐deliver these two drug molecules based on small molecule self‐assembly for breast cancer immunotherapy through synergizing oxidation therapy and epigenetic modulation (**Figure** **1**). The nanodrugs were composed of three small molecules, among which IR783 is a commercially available near‐infrared (NIR) dye with a heptamethine cyanine structure and serves as the stabilizer to form a nanoformulation.^[^ ^16^ ^]^ After intravenous injection, IR783‐stabilized nanodrugs could accumulate in tumor tissues based on passive targeting and be internalized into breast cancer cells through caveolin‐1 (CAV‐1)‐mediated endocytosis. The released *β*‐lap would generate a large amount of ROS in an NQO1‐dependent manner, which further oxidized IR783 into small fragments to promote nanoparticle disassembly and drug release. Moreover, CUDC101 could inhibit the bioactivities of EGFR and HDAC to regulate oncogenic transcription and synergize with *β*‐lap‐initiated oxidation therapy. Such a combinational strategy would elicit a strong ICD effect to secret DAMPs and tumor‐specific antigens for enhanced antitumor immune response. IR/Lap/CUDC NPs not only induced direct anticancer effects in primary tumors but also suppressed distant tumor growth by activating cytotoxic T cell and effector memory T cell response in the bilateral tumor‐bearing mouse model. This strategy presented novel insights into HDAC inhibition‐enhanced oxidation therapy and breast cancer immunotherapy, which were integrated into a simple and stable IR783‐stabilized nanoformulation with the potential for future clinical translation.

## 2 Results and Discussion

### 2.1 Preparation and Characterization of IR783‐Stabilized Nanodrugs

**Figure 2:**
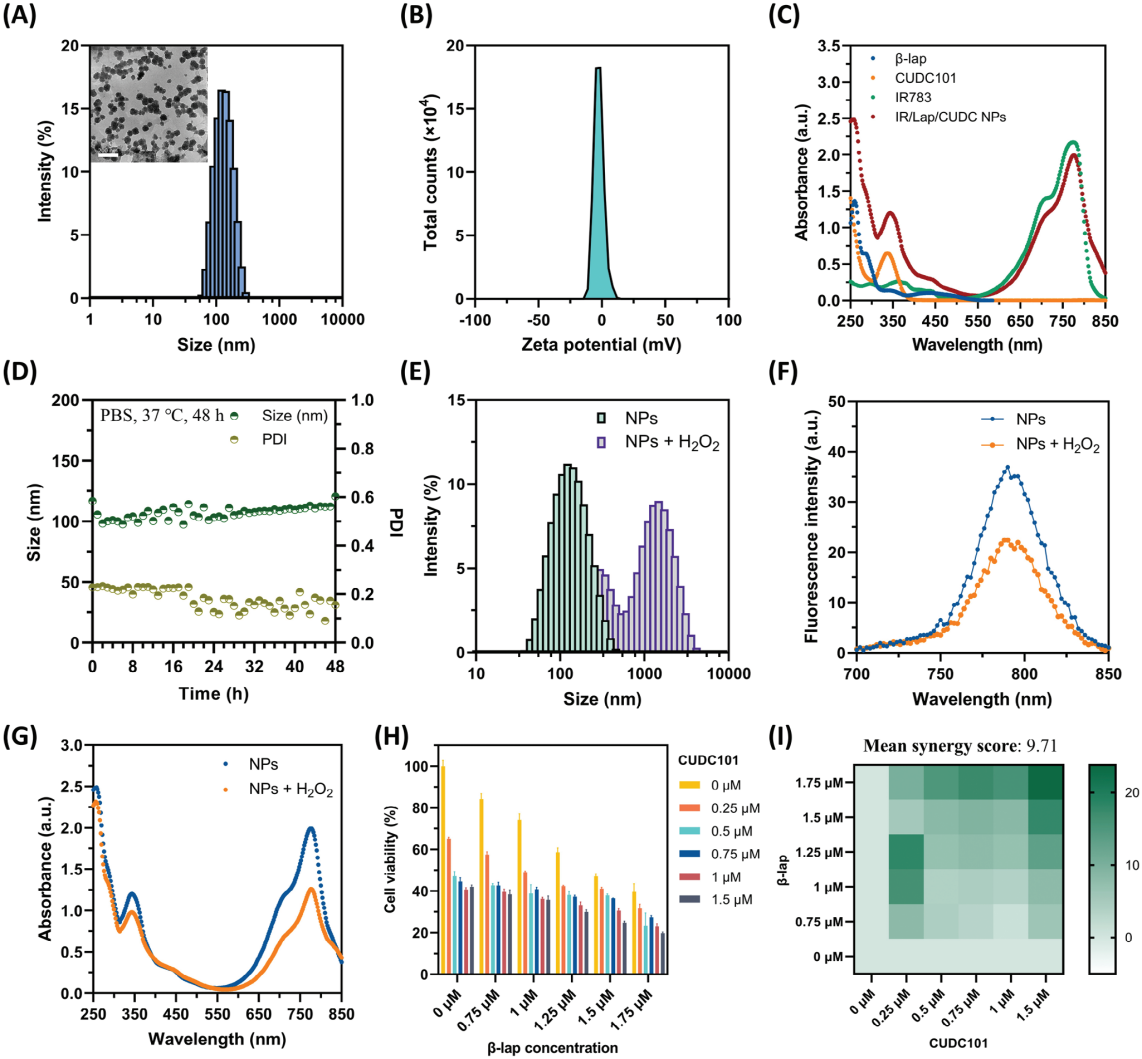
Preparation and characterization of IR/Lap/CUDC NPs. A) Size distribution and transmission electron microscopy (TEM) image of IR/Lap/CUDC NPs (Scale bar: 200 nm). B) Zeta potential of IR/Lap/CUDC NPs. C) UV–vis absorption spectra of free *β*‐lap, CUDC101, IR783, and IR/Lap/CUDC NPs. D) Stability test of IR/Lap/CUDC NPs under physiological conditions for 48 h. E) Size distribution changes of IR/Lap/CUDC NPs after H_2_O_2_ incubation. F) Fluorescent spectra of IR/Lap/CUDC NPs with or without H_2_O_2_ incubation. G) UV–vis absorption spectra of IR/Lap/CUDC NPs with or without H_2_O_2_ incubation. H) Cell viability of 4T1 cells treated with gradient concentrations of free *β*‐lap and CUDC101. I) Synergy score of free *β*‐lap and CUDC101 in 4T1 cells at gradient concentrations calculated by the Highest Single Agent (HSA) model in SynergyFinder. "NPs" shown in (E), (F), and (G) refers to IR/Lap/CUDC NPs.

Since both *β*‐lap and CUDC101 with potential antitumor effects are highly lipophilic and have poor pharmacokinetics, nanoparticle formulations may be a good choice to increase their water solubility and extend circulation time in blood vessels for better efficacy. Our previous work demonstrated that IR783, a NIR dye with a cyanine skeleton, could act as a stabilizer and self‐assemble with hydrophobic drugs to form nanoparticles.^[^ ^17^ ^]^ Therefore, we tried to utilize IR783 to fabricate a nanoformulation and co‐deliver *β*‐lap and CUDC101 into tumor tissues. IR/Lap/CUDC NPs were prepared by flash nanoprecipitation method. In brief, a mixed DMSO solution containing *β*‐lap and CUDC101 was pipetted into the IR783 aqueous solution quickly under a vigorous vortex. The unloaded drug molecules and excessive IR783 dye were removed by ultrafiltration, and IR/Lap/CUDC NPs were redistributed in deionized water. The feeding ratio of two drug molecules and the concentration of IR783 solution were screened first to optimize the formulation (Figure S1, Supporting Information). The results showed IR783 could successfully form nanoparticles with two drug molecules at a feeding ratio of 0.5 and a concentration of 50 µg mL^−1^. The hydrodynamic diameter of IR/Lap/CUDC NPs was ≈126.3 nm with a small polydispersity index (PDI) of ≈0.18 (**Figure** **2**A). Under transmission electron microscopy (TEM) imaging, IR/Lap/CUDC NPs exhibited spherical and well‐dispersed morphology. The zeta potential of the nanoparticles was ≈−4.44 mV, which might be attributed to the two negatively charged sulfonate groups in the IR783 skeleton (Figure 2B).

Afterward, we investigated the stability of IR/Lap/CUDC NPs in phosphate buffer saline (PBS) under physiological conditions. The results indicated that the nanoparticles could keep stable at 37 °C for at least 48 h (Figure 2D). From the UV–vis absorption spectra, IR/Lap/CUDC NPs exhibited strong absorption peaks at 260 nm (peak of *β*‐lap), 340 nm (peak of CUDC101), and 780 nm (peak of IR783), implying the successful encapsulation of two hydrophobic drug molecules (Figure 2C). Moreover, it was observed that the nanoparticles displayed a slight redshift at 800 nm, presumably attributed to the hydrophobic and *π*–*π* stacking interactions between IR783 and drug molecules. The encapsulation efficiency and loading capacity under different feeding ratios were determined by high‐performance liquid chromatography (HPLC). With the increase in the ratio of *β*‐lap to CUDC101, the encapsulation efficiency of CUDC101 was in the range of 40%–45%, while that of *β*‐lap decreased from 60% to 40% (Figure S2, Supporting Information). The loading capacity of CUDC101 decreased from 80% to 55%, while that of *β*‐lap increased from 10% to 37%. At the feeding ratio of 0.5, the encapsulation efficiency of *β*‐lap and CUDC101 was 56% and 44%, respectively. The calculated loading capacity of IR783, *β*‐lap, and CUDC101 was 8%, 37%, and 55%, respectively, indicating that two drug molecules were main components of IR/Lap/CUDC NPs in the presence of a small amount of IR783 as the stabilizer.

### 2.2 ROS‐Responsiveness of IR783‐Stabilized Nanodrugs

Since IR783 is sensitive to ROS and can be oxidized into smaller fragments, we next tried to explore the ROS‐responsiveness of IR/Lap/CUDC NPs. Hydrogen peroxide (H_2_O_2_) was chosen as the representative ROS for characterization experiments due to its relatively good stability and long half‐life in contrast to other kinds of ROS.^[^ ^18^ ^]^ After 0.5 h incubation of H_2_O_2_ (10 mm) at 37 °C, the particle size of IR/Lap/CUDC NPs increased to ≈700 nm with a higher PDI value ≈0.43, indicating the ROS‐responsive disassembly of the nanoparticles and the formation of large aggregates (Figure 2E). In normal conditions, IR/Lap/CUDC NPs exhibited strong fluorescence emission in the NIR region upon 650 nm excitation, which significantly decreased after H_2_O_2_ treatment (Figure 2F). The UV–vis absorption peak of the nanoparticles at 780 nm showed a similar trend (Figure 2G). In addition, consistent with the dynamic light scattering results, IR/Lap/CUDC NPs after H_2_O_2_ incubation became large irregular aggregates under TEM imaging (Figure S3, Supporting Information). These results suggested that the structure of IR783‐stabilized nanoparticles could be destroyed and form aggregates under oxidative conditions. Next, we investigated the drug release profiles under different conditions, and the results showed that the release amounts of *β*‐lap and CUDC101 after H_2_O_2_ incubation for 4 h were 1.21 and 1.46 times those under normal conditions, respectively, implying that H_2_O_2_ could promote drug release from the nanoparticles (Figure S4, Supporting Information). To explore the mechanism of ROS‐responsiveness, liquid chromatography‐mass spectrometry (LC‐MS) was used to analyze the residues of IR/Lap/CUDC NPs after H_2_O_2_ treatment. It was observed that the cyanine structure of IR783 underwent oxidative reactions and then degraded into small residues, which was consistent with the previous report (Figure S5, Supporting Information).^[^ ^17^ ^]^ Since IR783 played a critical role in the formation of the nanoparticles, its degradation under oxidative conditions might be the main reason for significant size change and improved drug release. Considering the higher ROS level in the tumor tissues of breast cancer patients and the potential ROS generation ability of the loaded *β*‐lap, such ROS‐responsiveness of IR/Lap/CUDC NPs may further facilitate the controlled drug release in cancer cells.^[^ ^19^ ^]^

### 2.3 Synergistic Effect Between *β*‐lap and CUDC101

It has been reported that *β*‐lap can generate ROS to exert cytotoxicity via specific biochemical reactions in cancer cells, while CUDC101 can inhibit tumor growth by multiple inhibitory effects on EGFR as well as HDAC signaling pathways.^[^ ^12^, ^20^ ^]^ We hypothesized that HDAC inhibition and epigenetic modulation effects caused by CUDC101 might amplify *β*‐lap‐induced oxidative stress and further promote DNA damage in breast cancer cells. To test this, we first explored the synergistic antitumor effect between *β*‐lap and CUDC101 in 4T1 cells, a mouse breast cancer cell line. The 4T1 cells were incubated with gradient concentrations of *β*‐lap and CUDC101, and cell viability was determined by MTT assay. Interestingly, we found that combinational treatment of two drug molecules exhibited robust synergistic inhibition effects compared to single drug groups (Figure 2H). For example, the cell viability was ≈24% with the combination of 1.5 µm *β*‐lap and CUDC101, which was significantly lower than that with 1.5 µm *β*‐lap (47%) or CUDC101 (42%) alone. Besides, the synergy score was calculated using the SynergyFinder website based on the cell viability results, and the average score was 9.71, indicating that the combination of the two drug molecules could synergistically inhibit cancer cell proliferation (Figure 2I). This combinational effect also worked in MDA‐MB‐231 cells, a human TNBC cell line, with an average synergy score of 7.92 (Figure S6, Supporting Information). Although *β*‐lap and CUDC101 showed combinational cytotoxicity, it was still difficult to inject both of them intravenously into breast cancer patients due to poor solubility and targeting effect. IR783 could work as a stabilizer to form nanodrugs to address this issue. The impact of IR783 on 4T1 cells was also explored, and the cell viability results showed that IR783 had no significant cytotoxicity against 4T1 cells (Figure S7, Supporting Information). With IR783‐stabilized nanodrug formulation, we next tried to investigate its cytotoxicity and internalization behaviors for efficient drug delivery and cancer treatment.

### 2.4 Cytotoxicity and Internalization of IR/Lap/CUDC NPs

**Figure 3:**
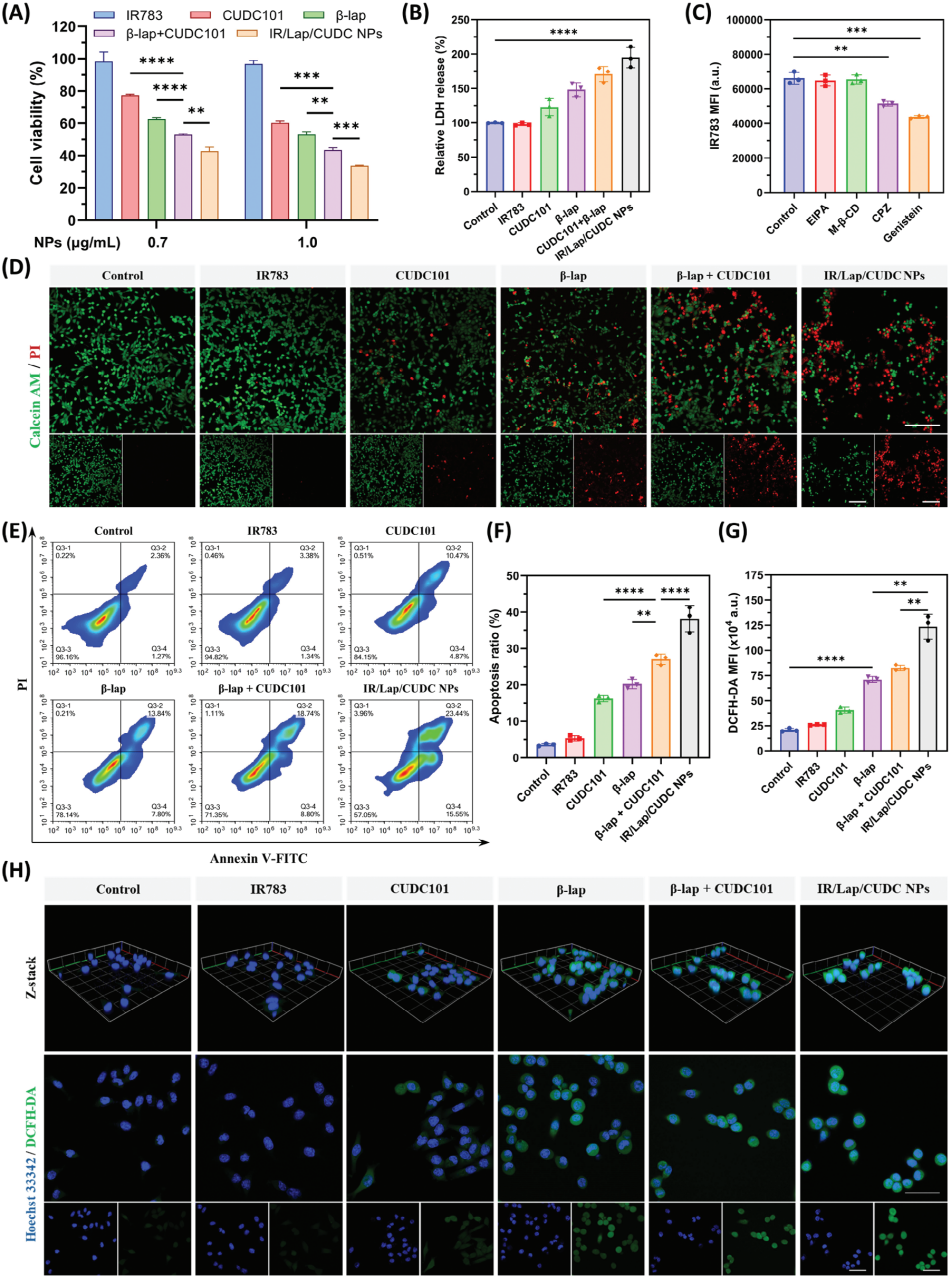
Cytotoxicity and ROS generation ability of IR/Lap/CUDC NPs. A) Cell viability of 4T1 cells treated with different formulations. B) Lactate dehydrogenase (LDH) release level of 4T1 cells after different treatments. C) Cellular uptake level of IR/Lap/CUDC NPs in 4T1 cells with different endocytosis inhibitors. D) Live and dead staining of 4T1 cells treated with different formulations (Scale bar: 200 µm). E) Apoptosis level of 4T1 cells treated with different formulations. F) Quantitative analysis of the apoptosis assay. G) Flowcytometric analysis of ROS generation in 4T1 cells. H) Representative confocal laser scanning microscopy (CLSM) images of ROS in 4T1 cells after different treatments (Scale bar: 50 µm; Grid side length in Z‐stack: 30 µm). Data were shown as mean ± SD. ^*^ *p* < 0.05, ^**^ *p* < 0.01, ^***^ *p* < 0.001, ^****^ *p* < 0.0001, ns (not significant).

To explore the cytotoxicity of IR/Lap/CUDC NPs, we first measured the cell viability of 4T1 cells treated with different concentrations of the nanoparticles. The results indicated that nanoparticle treatment could effectively suppress breast cancer cell growth with better efficacy in contrast to free drug groups (**Figure** **3**A). Moreover, nanodrugs could also promote lactate dehydrogenase (LDH) release from the cytosol compared with the control groups, suggesting its robust anticancer effect (Figure 3B). Regarding cancer cell death, both confocal imaging and flowcytometric analysis indicated that IR/Lap/CUDC NPs could efficiently induce cancer cell apoptosis, especially late apoptosis, which was 1.25 times that of the dual‐drug group (Figure 3D–F). Since *β*‐lap was reported to have ROS generation ability inside cancer cells, we next explored the intracellular total ROS levels after different treatments. Flowcytometric assay demonstrated that the nanoparticles could significantly elevate the intracellular ROS level in contrast to other groups (Figure 3G). Furthermore, the ROS generated after nanodrug treatment was localized in both the cytoplasm and the nucleus, as shown by Z‐stack multi‐layer imaging, suggesting its potential to increase oxidative stress in organelles and chromosomes, thereby promoting cell apoptosis (Figure 3H).

It was reported that *β*‐lap could first produce superoxide anion (·O_2_^−^) during the NQO1 catalytic cycle inside cancer cells, which further generated H_2_O_2_ with the help of superoxide dismutase.^[^ ^21^ ^]^ H_2_O_2_ and ·O_2_^−^ could react with intracellular iron ion or nitric oxide to produce hydroxyl radical (·OH) or peroxynitrite anion (ONOO^−^) with much higher oxidative reactivity, respectively.^[^ ^22^ ^]^ To investigate the types of ROS generated from IR/Lap/CUDC NPs after cell internalization, 4T1 cells were treated with different formulations and stained with dihydrorhodamine 123 (DHR 123), hydroxyphenyl fluorescein (HPF), and SOSG probes to detect the intracellular ·O_2_^−^, ·OH and ONOO^−^, and singlet oxygen (^1^O_2_) levels, respectively.^[^ ^23^ ^]^ The confocal imaging results showed that IR783‐stabilized nanodrug treatment could produce large amounts of ·O_2_^−^, ·OH, and ONOO^−^ inside 4T1 cells with a low ^1^O_2_ production level (Figure S8, Supporting Information). Besides, nanodrugs exhibited better ROS generation ability in contrast to free drug groups in terms of different types of ROS, which might contribute to their enhanced pro‐apoptotic effect.

The enhanced apoptosis and ROS generation ability inspired us to evaluate the cell internalization properties of IR/Lap/CUDC NPs. Compared with free IR783 dye group, the nanoparticles exhibited significantly higher intracellular fluorescent intensity after 1 or 4 h of incubation, indicating a higher level of cellular uptake in 4T1 cells (Figure S9, Supporting Information). To investigate the endocytosis pathways of nanodrugs, we pretreated 4T1 cancer cells incubated with different endocytosis inhibitors before nanodrug treatment. The flowcytometric analysis showed that chlorpromazine (CPZ) and genistein could significantly suppress the internalization of IR/Lap/CUDC NPs (Figure 3C). CPZ and genistein belong to inhibitors of clathrin and caveolin‐1 (CAV‐1)‐mediated endocytosis pathways, respectively.^[^ ^24^ ^]^ Hence, the nanoparticles mainly relied on these two endocytosis pathways to enter 4T1 cells. Previous studies reported that some breast cancer cells have high CAV‐1 expression levels and IR783‐incorporated nanoformulations presented CAV‐1 active targeting effect.^[^ ^16^, ^25^ ^]^ Similarly, such targeting effect based on IR783 might also contribute to the efficient cellular uptake of IR/Lap/CUDC NPs in our design. To conclude, IR783‐stabilized nanodrugs could effectively induce ROS generation and cancer cell apoptosis after clathrin and CAV‐1‐mediated endocytosis in 4T1 cells. Since the encapsulated *β*‐lap and CUDC101 have unique pharmacological effects in cancer therapy, we next focused on their bioactivities and the mechanism of synergistic performance.

### 2.5 NQO1‐Dependent ROS Generation Caused by IR/Lap/CUDC NPs

**Figure 4:**
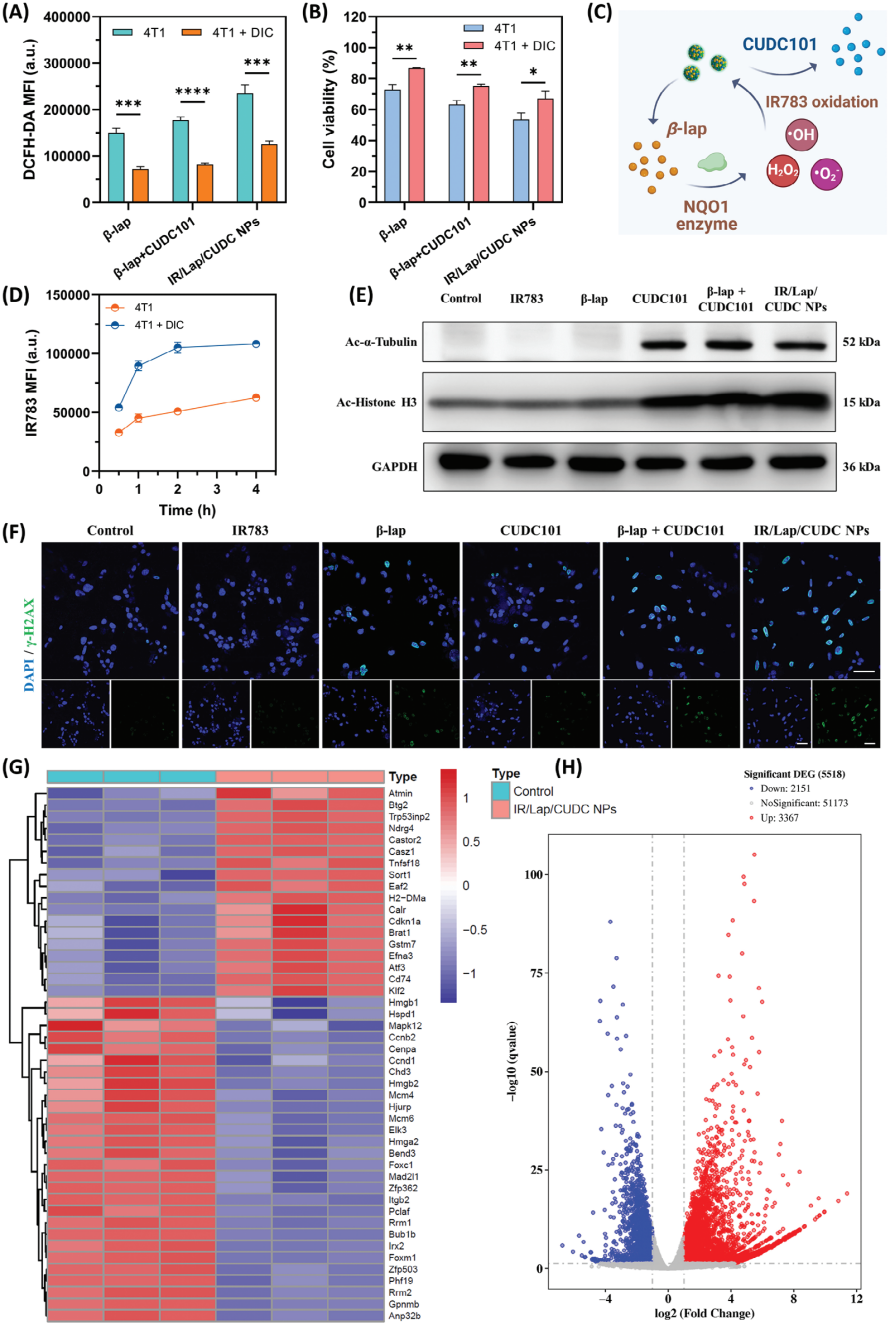
NQO1‐dependent ROS generation and HDAC inhibition caused by IR/Lap/CUDC NPs. A) Flowcytometric analysis of ROS generation in 4T1 cells after different treatments with or without dicoumarol (DIC). B) Cell viability of 4T1 cells after different treatments with or without DIC. C) Schematic illustration of NQO1‐dependent ROS generation and subsequent IR783 oxidation. D) Intracellular IR783 fluorescence in 4T1 cells treated with IR/Lap/CUDC NPs with or without DIC. E) Western blot analysis of acetylated α‐tubulin, acetylated histone H3, and GAPDH expression levels in 4T1 cells after different treatments. F) Representative CLSM images of γ‐H2AX in 4T1 cells after different treatments (Scale bar: 50 µm). G) Heatmap of specific DEGs between normal 4T1 cells and IR/Lap/CUDC NPs‐treated 4T1 cells. H) Volcano plot of total DEGs between normal 4T1 cells and IR/Lap/CUDC NPs‐treated 4T1 cells. Data were shown as mean ± SD. ^*^ *p* < 0.05, ^**^ *p* < 0.01, ^***^ *p* < 0.001, ^****^ *p* < 0.0001, ns (not significant).

To explore the mechanism of ROS generation, we used dicoumarol (DIC) as the NQO1 enzyme competitive inhibitor to pretreat 4T1 cancer cells.^[^ ^26^ ^]^ Both flowcytometric assay and confocal imaging indicated that DIC could significantly inhibit *β*‐lap‐induced ROS production inside 4T1 cells, possibly due to NQO1 inhibition after DIC treatment (**Figure** **4**A; Figure S10A, Supporting Information). It was worth noting that DIC pretreatment resulted in a reduction of 46.8% in total ROS in the nanoparticles group. Since oxidative stress plays a crucial role in cancer cell apoptosis, we conducted an apoptosis assay with or without DIC pretreatment. The results showed that DIC‐initiated NQO1 inhibition could suppress the pro‐apoptotic effects of IR/Lap/CUDC NPs, particularly the late‐stage apoptosis level, which decreased from 25.6% to 14.1% (Figure S10B,C, Supporting Information). The MTT assay showed a similar trend with higher cell viability after DIC pretreatment across different groups, suggesting that IR/Lap/CUDC NPs exhibited NQO1‐dependent ROS generation and cytotoxicity (Figure 4B). It has been reported that breast tumor tissues owned higher NQO1 enzyme expression levels compared with normal tissues.^[^ ^27^ ^]^ Therefore, such NQO1 expression difference may contribute to the selective killing of cancer cells.

The other consideration was the ROS responsiveness of the cyanine group in the IR783 skeleton. We hypothesized that increased ROS levels in the cytoplasm might lead to structural changes in IR783 and promote nanoparticle dissociation (Figure 4C). To test this, 4T1 cells with or without DIC treatment were incubated with IR/Lap/CUDC NPs, and the IR783 fluorescence was determined by flow cytometry at different time points. The results demonstrated that fluorescent intensity in the control group was much lower than that of the DIC treatment group, implying that elevated oxidative stress might cause IR783 degradation and the following disassembly of nanodrugs (Figure 4D). To further explore the ROS reaction ability of IR783, 4T1 cells were treated with *β*‐lap and different concentrations of IR783, and the intracellular ROS level was determined. With 0.1 µg mL^−1^ IR783, which was the same concentration of IR783 in nanodrug treatment, the ROS level was reduced by 6.78% in contrast to the *β*‐lap group. At a higher concentration, it was reduced by 14% (Figure S11, Supporting Information). Therefore, IR783 reacted with a small amount of ROS generated by *β*‐lap, which would not significantly affect the oxidation therapy due to the limited consumption of ROS. Besides, the cascade ROS response of IR/Lap/CUDC NPs could fully exploit NQO1‐dependent ROS production and subsequent oxidative reactions to promote drug release, which has the potential to achieve tumor‐targeting drug delivery and reduce side effects.

### 2.6 Epigenetic Modulation and Anti‐Migration Effects

Apart from *β*‐lap‐provoked oxidation therapy, the multiple inhibitory effects from CUDC101 also contributed a lot to the synergistic cooperation. CUDC101 was designed using a combined chemical structure of hydroxamic acid and quinazoline, endowed with HDAC and EGFR dual inhibitory effects.^[^ ^12a^ ^]^ We first investigated its HDAC inhibitory function by western blot analysis in 4T1 cells. The results indicated that IR/Lap/CUDC NPs could significantly increase the acetylation levels of α‐tubulin and histone H3, suggesting the low bioactivity of HDAC enzymes after nanodrug treatment (Figure 4E; Figure S12, Supporting Information). Since histone deacetylation caused by HDAC enzymes was reported to compress chromatin structure and promote tumorigenesis through epigenetic regulation, CUDC101‐initiated HDAC inhibition might loosen chromatin structure and suppress the oncogenic signaling pathways to synergize with *β*‐lap for combinational anticancer effect.^[^ ^13a^ ^]^ In parallel, the elevated histone acetylation level could neutralize the positive charges of histones and reduce their electrostatic interactions with nuclear DNA, which might further enhance DNA damage in oxidation therapy. Therefore, we next detected the γ‐H2AX level in the nucleus by confocal imaging to evaluate DNA damage (Figure 4F). The results demonstrated that *β*‐lap together with CUDC101 exhibited more severe DNA double‐strand break and higher γ‐H2AX levels compared with single drug groups, implying their synergistic effect on DNA damage (Figure S13, Supporting Information). Moreover, IR/Lap/CUDC NPs treatment showed better DNA damage effects compared to the free drug groups, possibly attributed to higher cellular uptake levels and ROS generation ability.

In addition to epigenetic regulation, the other pharmacological action of CUDC101 serves as an EGFR tyrosine kinase inhibitor, suppressing proliferation and migration. Since tumor cell metastasis is closely related to poor prognosis in breast cancer patients, scratch and transwell assays were conducted to determine the lateral and vertical migration abilities of 4T1 cells after nanodrug treatment (Figures S14 and S15, Supporting Information). The quantitative results revealed that IR/Lap/CUDC NPs displayed a stronger anti‐migration effect compared to the control groups. Collectively, IR783‐stabilized nanodrugs exhibited synergistic anticancer effects based on ROS production and epigenetic modulation, which could further induce DNA damage and prevent cell migration, contributing to effective breast cancer treatment.

To investigate the downstream molecular mechanisms, transcriptome sequencing analysis of total RNA from different samples was performed. Among 56691 genes examined, 3367 genes were up‐regulated (red dots) while 2151 genes were down‐regulated (blue dots) in the 4T1 cells treated with IR/Lap/CUDC NPs compared to the control group (Figure 4H). Gene ontology (GO) enrichment analysis was conducted to determine the differences in biological functions. The results showed that nanodrug treatment could influence DNA binding, chromatin binding, transcription, and apoptotic processes (Figure S16, Supporting Information). Additionally, the Kyoto Encyclopedia of Genes and Genomes (KEGG) enrichment analysis demonstrated that the differentially expressed genes (DEGs) were highly enriched in glutathione metabolism, peroxisome (oxidative stress), cell cycle, MAPK signaling pathway (HDAC inhibition), and cell adhesion molecules (EGFR inhibition) (Figure S17, Supporting Information). Regarding specific DEGs, the transcription of Ndrg4, Sort1, Klf2, and Btg2, which participate in the regulation of oxidative stress‐induced apoptosis, was significantly up‐regulated in the IR/Lap/CUDC NPs group (Figure 4G). Moreover, the high expression levels of H2‐DMa and Calr suggested that the nanodrugs might promote antigen presentation and antitumor immunity. In addition, Rrm1 and Rrm2 related to DNA replication and repair were down‐regulated, suggesting DNA damage after nanodrug treatment. Furthermore, the expression levels of five migration‐related genes, including Mapk12, Itgb2, Foxc1, Elk3, and Gpnmb, were also decreased. Consistent with the in vitro studies, these findings collectively indicate that IR/Lap/CUDC NPs could effectively suppress breast cancer cell growth through oxidation therapy and epigenetic regulation.

### 2.7 Immunogenic Cell Death and Pro‐Inflammatory Effects

**Figure 5:**
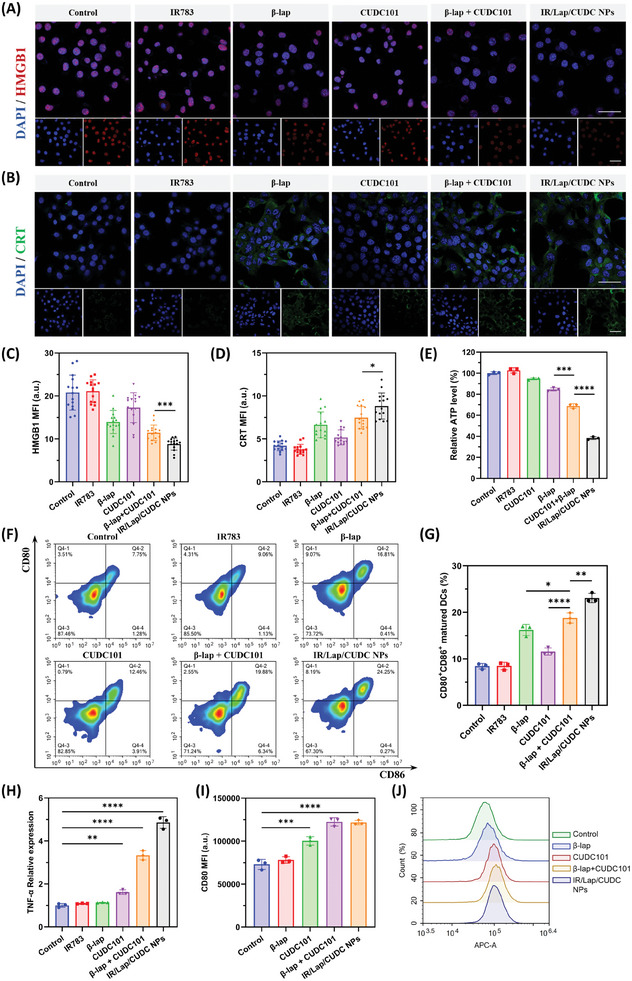
Immunogenic cell death and pro‐inflammatory effects of IR/Lap/CUDC NPs. A) Representative CLSM images of HMGB1 in 4T1 cells after different treatments (Scale bar: 50 µm). B) Representative CLSM images of CRT in 4T1 cells after different treatments (Scale bar: 50 µm). C) Quantitative analysis of HMGB1 confocal imaging assay. D) Quantitative analysis of CRT confocal imaging assay. E) Relative intracellular ATP level of 4T1 cells treated with different formulations. F) Flowcytometric analysis of bone marrow‐derived dendritic cells (BMDCs) maturation after incubation with the supernatant of 4T1 cells treated with different formulations. G) Quantitative analysis of CD80^+^CD86^+^ BMDCs maturation level. H) mRNA expression of TNF‐α in RAW264.7 cells after different treatments. I) Quantitative analysis of CD80 expression level in RAW264.7 cells. J) Flowcytometric analysis of CD80 expression level in RAW264.7 cells after different treatments. Data were shown as mean ± SD. ^*^ *p* < 0.05, ^**^ *p* < 0.01, ^***^ *p* < 0.001, ^****^ *p* < 0.0001, ns (not significant).

Some cancer patients are insensitive to standard immunotherapies due to intrinsic immunosuppressive microenvironment, which is characterized as a cold tumor.^[^ ^28^ ^]^ One of the solutions to reverse cold tumors into immunologically hot tumors is sufficient antigen release and pro‐inflammatory cytokines secretion. Recent studies have reported that *β*‐lap can initiate ICD via oxidative stress to generate sufficient tumor antigens released from dying cancer cells.^[^ ^8a^ ^]^ To explore the antitumor immune response of IR/Lap/CUDC NPs, we conducted confocal imaging to detect the protein expression levels of calreticulin (CRT) and high mobility group protein 1 (HMGB1), both of which belong to characteristic markers during ICD. Compared to free drug groups, nanodrugs showed the lowest level of HMGB1 in the nucleus (**Figure** **5**A,C). Besides, nanodrugs could also upregulate CRT expression level on the cell membrane, suggesting the strong immune activation ability of IR/Lap/CUDC NPs (Figure 5B,D). Another ICD hallmark is the adenosine triphosphate (ATP). Intracellular ATP concentration was determined by luminescence assay, and the results showed that the ATP level in the nanodrugs group was dramatically decreased to 40% compared to the control group (Figure 5E). Surface CRT molecules can serve as an “eat me” signal while extracellular HMGB1 and ATP can function as a “danger signal”, all of which would activate dendritic cells to promote antigen presentation. To test this, bone marrow‐derived dendritic cells (BMDCs) were collected from mice and incubated with the supernatants of 4T1 cells after different treatments. The flowcytometric results indicated that the nanodrugs group could significantly promote CD80^+^CD86^+^ matured dendritic cell ratio, indicating the strong ICD effect of IR/Lap/CUDC NPs to release sufficient antigens, activate BMDCs, and stimulate immune response (Figure 5F,G).

Apart from antigen release, tumor‐associated macrophages (TAMs) also play an essential role in anticancer immune response. However, most TAMs in the microenvironment tend to be polarized into tumor‐promoting phenotypes with anti‐inflammatory properties to help the surviving tumor cells and restrict immunotherapy.^[^ ^29^ ^]^ It was reported that HDAC inhibition and epigenetic modulation might re‐educate immunosuppressive TAMs and facilitate pro‐inflammatory cytokines release.^[^ ^12^, ^13^, ^30^ ^]^ To test this, RAW264.7 macrophage cells were first induced to tumor‐promoting phenotype with murine IL‐4 and IL‐13 to mimic TAMs. After nanodrug treatment, qPCR analysis showed that the gene expression levels of pro‐inflammatory cytokines, such as TNF‐α and IL‐1β, were significantly increased compared to the control group (Figure 5H; Figure S18, Supporting Information). Besides, IR/Lap/CUDC NPs could also upregulate the expression level of CD80 protein, a necessary co‐stimulatory molecule for T cell proliferation, on the cell membrane of macrophages (Figure 5I,J). These results suggested that IR783‐stabilized nanodrugs could promote pro‐inflammatory cytokine gene expression in the macrophages, which might further facilitate T cell recruitment and infiltration as well as boost anticancer immune response.

### 2.8 Biodistribution and Antitumor Efficacy of IR/Lap/CUDC NPs

**Figure 6:**
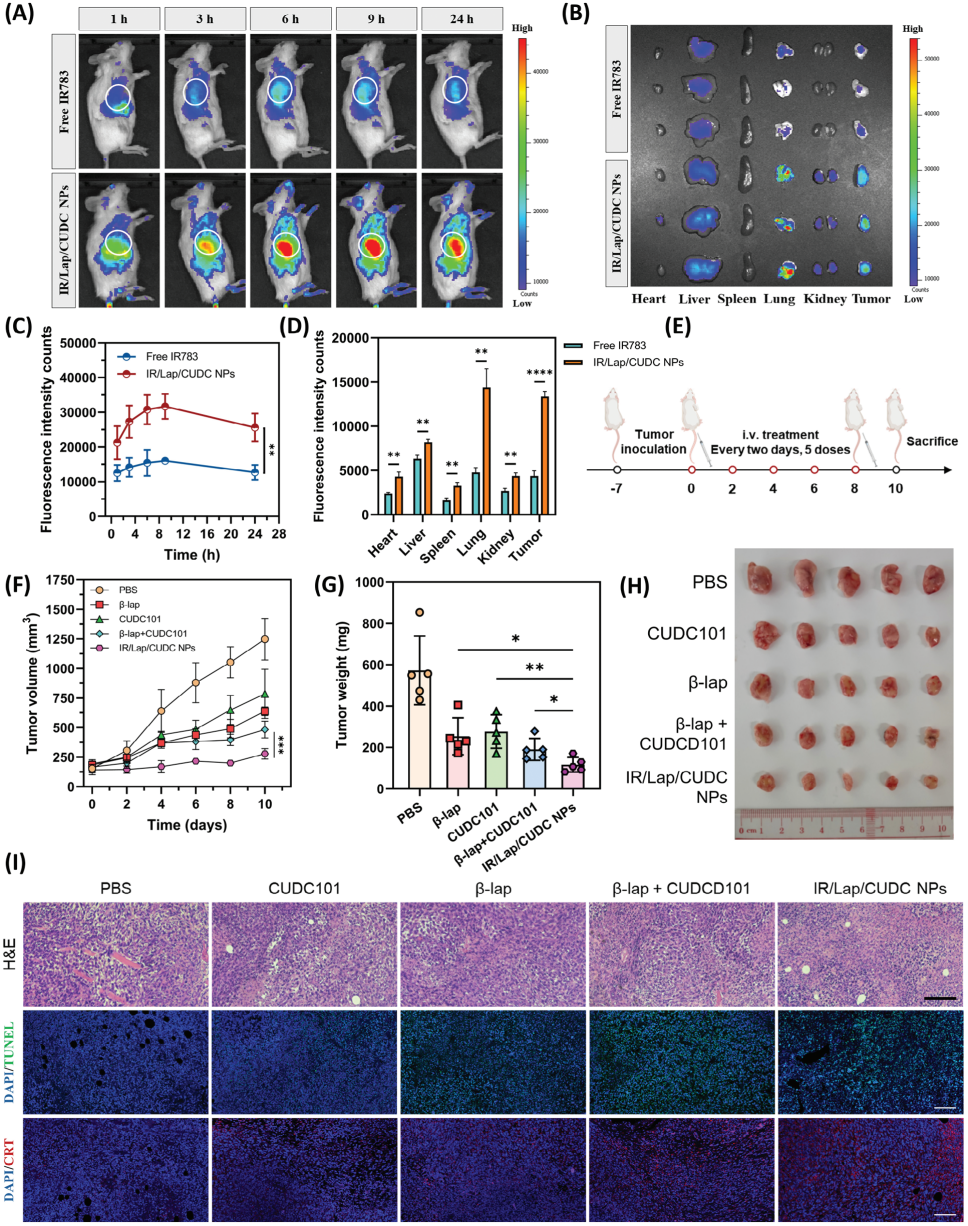
Biodistribution and antitumor growth effect of IR/Lap/CUDC NPs. A) Fluorescence imaging and biodistribution of free IR783 and IR/Lap/CUDC NPs in the 4T1 tumor‐bearing BALB/c mouse model (*n* = 3). B) Ex vivo fluorescence image of tumors and main organs collected from the mice treated with free IR783 or IR/Lap/CUDC NPs after 24 h. C) Fluorescence intensity within the tumor area at different time points after the intravenous injection of IR783 or IR/Lap/CUDC NPs. D) Quantitative analysis of the fluorescence intensity in tumor tissues and main organs collected from the treated mice. E) Therapeutic schedule using IR/Lap/CUDC NPs for oxidation therapy and epigenetic modulation against 4T1 subcutaneous tumors. F) Tumor volume change of the mice in different treatment groups during the 10‐day therapeutic period (*n* = 5). G) Tumor weight of the mice in different treatment groups on day 10. H) The image of tumors collected from the treated mice at day 10. I) Hematoxylin and eosin (H&E) staining (Scale bar: 200 µm), TUNEL assay (Scale bar: 100 µm), and CRT immunofluorescence imaging (Scale bar: 100 µm) of the tumor tissues from different treatment groups. Data were shown as mean ± SD. ^*^ *p* < 0.05, ^**^ *p* < 0.01, ^***^ *p* < 0.001, ^****^ *p* < 0.0001, ns (not significant).

To explore the applicability of a synergistic antitumor strategy combining oxidation therapy and epigenetic modulation, a 4T1 tumor‐bearing BALB/c mouse model was established. The in vivo imaging system (IVIS) experiment indicated that IR/Lap/CUDC NPs exhibited higher tumor retention ability compared to the free IR783 dye at different time points after systemic administration (**Figure** **6**A,C). The fluorescence within the main organs and tumor tissues was measured ex vivo 24 h after injection, and the results demonstrated that fluorescence intensity of nanodrugs in tumor tissues was 3.05 times that of the free IR783 group, possibly attributed to the extended circulation time and CAV‐1‐based targeting effect (Figure 6B,D). It should be noted that due to the ROS generation and IR783 oxidation within tumor tissues, the measured accumulation level of the nanodrugs based on the intratumoral fluorescence intensity of IR783 might be lower than that in the real situation. Nevertheless, it could be concluded that IR783‐stabilized nanodrugs mainly accumulated in the tumor, lungs, and liver, with less accumulation in other organs, implying our designed nanoformulation could deliver drug molecules into tumor tissues. After that, we investigated the antitumor efficacy of IR/Lap/CUDC NPs in the subcutaneous 4T1 breast cancer model (Figure 6E). The nanodrugs demonstrated a better inhibition effect on tumor volume compared to the free drug groups (Figure 6F; Figure S19, Supporting Information). Particularly, the dual‐drug group exerted a combinational anticancer effect from oxidation therapy and epigenetic modulation, which was consistent with the in vitro studies. On day 10, tumor tissues were collected and photographed. The results showed that IR/Lap/CUDC NPs could effectively suppress tumor growth, resulting in the lowest tumor weight (Figure 6G,H). In addition, H&E staining and TUNEL assay revealed significant tumor tissue damage after nanodrug treatment (Figure 6I). Collectively, these findings verified that IR783‐stabilized nanodrugs could elicit a durable therapeutic response with considerable antitumor efficacy.

During the treatment, systemic toxicity and obvious side effects of different formulations were not observed with minimal morphological changes in H&E staining of the main organs as well as negligible body weight fluctuation (Figures S20 and S21A, Supporting Information). Besides, liver functional assays, such as alanine transaminase (ALT) and aspartate transaminase (AST), showed no significant difference among different groups (Figure S21B,C, Supporting Information). These results suggested the biosafety of the nanodrugs in the tumor‐bearing mouse model with no severe adverse effects observed. It is worth noting that *β*‐lap and CUDC101 are under clinical trials as antitumor drug candidates (NCT01171924, NCT00358930), and IR783 is a well‐established NIR fluorescent dye widely used in preclinical and clinical studies. Given these factors, our IR/Lap/CUDC NPs, with their simple design and stable structure, hold great potential for future clinical translation, offering a safe and efficient approach to breast cancer treatment.

### 2.9 Antitumor Immune Activation and Distant Tumor Inhibition Effects

**Figure 7:**
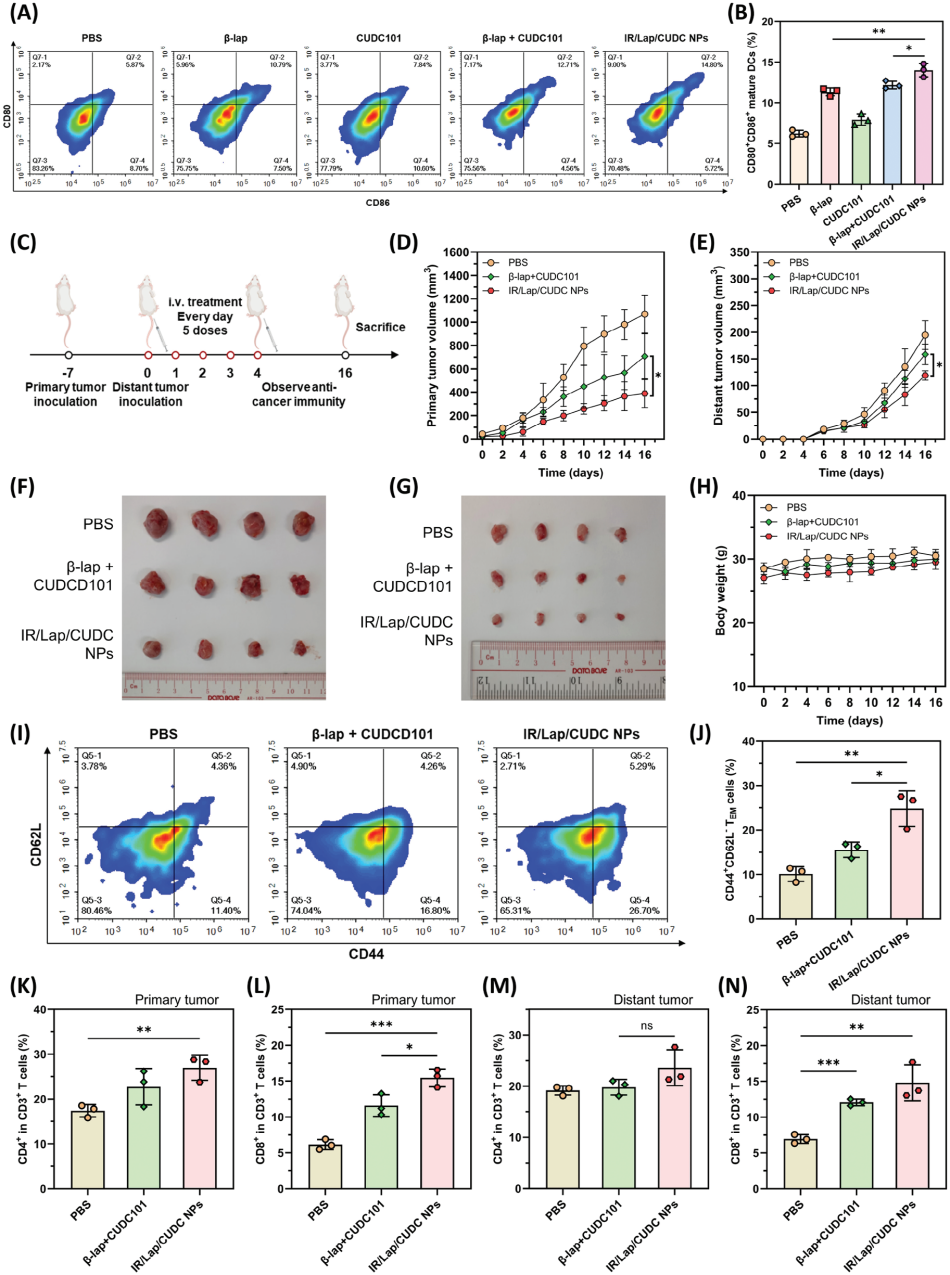
Antitumor immune activation and distant tumor inhibition effect of IR/Lap/CUDC NPs. A) Flowcytometric analysis of dendritic cell maturation in the tumor‐draining lymph nodes collected from the treated mice. B) Quantitative results of CD80^+^CD86^+^ matured dendritic cells of different groups. C) Therapeutic schedule using IR/Lap/CUDC NPs for anticancer immune response and distant tumor inhibition. D) Primary tumor growth profiles of the treated mice (*n* = 4). E) Distant tumor growth profiles of the treated mice (*n* = 4). F) The image of primary tumors collected from the tumor‐bearing mice on day 16. G) The image of distant tumors collected from the tumor‐bearing mice on day 16. H) Body weight change of the tumor‐bearing mice with different treatments. I) Flowcytometric analysis of effector memory T cells in the spleen collected from the treated mice. J) Quantitative results of CD44^+^CD62L^−^ effector memory T cells of different groups. K) The tumor‐infiltrating CD4^+^ T cells in total CD3^+^ T cells in primary tumor tissues. L) The tumor‐infiltrating CD8^+^ T cells in total CD3^+^ T cells in primary tumor tissues. M) The tumor‐infiltrating CD4^+^ T cells in total CD3^+^ T cells in distant tumor tissues. N) The tumor‐infiltrating CD8^+^ T cells in total CD3^+^ T cells in distant tumor tissues. Data were shown as mean ± SD. ^*^ *p* < 0.05, ^**^ *p* < 0.01, ^***^ *p* < 0.001, ^****^ *p* < 0.0001, ns (not significant).

Given that IR/Lap/CUDC NPs can elicit ICD and pro‐inflammatory effects through oxidation therapy and epigenetic modulation in vitro, we proposed that such antitumor immune response might contribute to the cancer immunotherapy to reshape immunosuppressive microenvironment in vivo. The immunofluorescence assay of tumor tissue sections revealed that the nanodrug treatment resulted in the highest CRT protein expression level compared to the free drug groups, indicating a strong immune stimulation effect (Figure 6I). It was reported that CRT exposure would act as a kind of DAMP to activate dendritic cell maturation and antigen presentation.^[^ ^31^ ^]^ Therefore, the tumor‐draining lymph nodes (TDLNs) were harvested after the endpoint. The flowcytometric analysis indicated that the IR/Lap/CUDC NPs group showed a higher percentage of CD80^+^CD86^+^ cells in CD45^+^MHC‐II^+^CD11c^+^ immune cells, implying the elevated dendritic cell maturation level in the TDLNs (**Figure** **7**A,B). With sufficient antigen release and presentation, naïve T cells can be stimulated to infiltrate tumor tissues and exert cell‐killing effects. To explore the systemic T cell response and immunological memory against cancer cells, a distant 4T1 tumor model was established seven days after primary tumor inoculation (Figure 7C). After daily treatment from day 0 to day 4, there was no treatment, and the volume of primary and distant tumors was recorded every two days to observe anticancer immune response. For the primary tumors, we observed a similar trend with the lowest tumor volume and weight in the nanodrugs group (Figure 7D,F). For the distant tumors, they could mimic breast cancer metastasis and reflect systemic antitumor immunity.^[^ ^32^ ^]^ The results suggested that IR/Lap/CUDC NPs could also suppress distant tumor growth with better efficacy compared to the control groups (Figure 7E,G). In parallel, the tumor weight results showed a similar trend (Figure S22, Supporting Information). Negligible body weight fluctuation was observed during the therapeutic period. These findings verified the distant tumor inhibition ability of IR783‐stabilized nanodrugs without obvious adverse effects (Figure 7H).

To investigate the underlying mechanism of anticancer immune response, spleens were collected from the bilateral 4T1 tumor‐bearing mice on day 16. Flowcytometric analysis revealed that the percentage of CD44^+^CD62L^−^ effector memory T (T_EM_) cells in CD3^+^CD8^+^ T cells were elevated from 11.4% to 16.8% by free‐drug combination, further increased to 26.7% in the nanodrugs group (Figure 7I,J). Given that T_EM_ cells play a critical role in preventing tumor metastasis and recurrence, it can be inferred that IR/Lap/CUDC NPs could elicit considerable immune memory effects for enhanced immunotherapy.^[^ ^33^ ^]^ The other consideration was remodeling the tumor immunosuppressive microenvironment. It is worth noting that nanodrugs could significantly elevate the proportion of CD80^+^ TAMs in CD45^+^F4/80^+^ immune cells in primary tumor tissues with decreased CD206^+^ anti‐inflammatory TAMs population, implying their pro‐inflammatory and polarizing effects in tumor microenvironment to reshape the cold tumors (Figure S23, Supporting Information). Moreover, the response of tumor‐infiltrating T cells in primary and distant tumors was explored as well (Figures S24 and S25, Supporting Information). Compared to the control group, IR/Lap/CUDC NPs resulted in a significantly higher percentage of infiltrating CD4^+^ helper T lymphocytes and CD8^+^ cytotoxic T lymphocytes in CD45^+^CD3^+^ T cells in the primary tumor tissues (Figure 7K,L). Likewise, the distant tumor results showed similar trends with increased CD8^+^ cytotoxic T‐cell infiltration levels in the nanodrugs group (Figure 7M,N). Collectively, these flowcytometric analyses demonstrated that the co‐delivery of *β*‐lap and CUDC101 through IR783‐stabilized nanoformulation exhibited amplified cytotoxic T cell response and systemic anticancer immune activation to inhibit both primary and distant tumor growth effectively.

The designed IR/Lap/CUDC NPs are composed of *β*‐lap as a ROS inducer, CUDC101 as an EGFR and HDAC dual inhibitor, and IR783 as a stabilizer, which integrate oxidation therapy with epigenetic regulation to provide efficient breast cancer immunotherapy. The HDAC inhibition and epigenetic modulation effects induced by CUDC101 could amplify the *β*‐lap‐initiated oxidative stress and further promote DNA damage and cancer cell apoptosis in a synergistic manner, thus leading to sufficient antigen release and an anticancer immune response. Additionally, IR783 could help form a nanodrug formulation with good stability and size distribution, facilitate tumor‐targeting delivery through the enhanced permeability and retention effect as well as the CAV‐1‐mediated endocytosis pathway, and achieve self‐amplified, ROS‐responsive nanoparticle dissociation and drug release in breast cancer cells. In terms of clinical translation, the IR783‐stabilized nanodrug system could benefit industrial production due to its simple and stable formulation, which can be achieved using microfluidics to mix the three components. However, this strategy still faces some challenges. The ROS generation ability of nanodrugs is dependent on the NQO1 enzyme expression level. Therefore, the effects of oxidation therapy and ROS‐responsive drug release may vary between individuals. In addition, the feed ratio of the three components needs to be optimized during large‐scale production to ensure a suitable size distribution and a good combinational effect. Lastly, since the cyanine structure in IR783 is easily oxidized, the fabricated nanodrugs need to be protected from air during transportation and long‐term storage.

## 3 Conclusion

In summary, our results provided strong evidence for the synergistic potential of combining *β*‐lap‐mediated oxidation therapy and CUDC101‐provoked epigenetic modulation for breast cancer treatment. To co‐deliver two hydrophobic drug molecules, an IR783‐stabilized nanodrug formulation was designed with a simple and stable structure and high drug loading capacity. The nanodrugs could be internalized into breast cancer cells through both clathrin and CAV‐1‐mediated endocytosis and generate ROS in an NQO1‐dependent manner. Furthermore, the ROS generation would promote drug release and synergize with HDAC inhibition to cause severe DNA damage, cancer cell apoptosis, and a strong ICD effect, thereby leading to tumor antigen release and dendritic cell maturation. In the tumor‐bearing mouse model, IR/Lap/CUDC NPs were shown to accumulate in tumor tissues, inhibit both primary and distant tumor growth, and boost antitumor immunity to reshape immunosuppressive tumor microenvironment for efficient breast cancer immunotherapy. This multifunctional and biocompatible IR783‐stabilized nanosystem offers a promising strategy for HDAC inhibition‐enhanced oxidation therapy and holds the potential for clinical translation and cancer treatment in the future.

## 4 Experimental Section

### Materials

*β*‐lapachone (*β*‐lap), crystal violet, and DNase I were obtained from Macklin (Shanghai, China). CUDC101, hydroxyphenyl fluorescein (HPF), dihydrorhodamine 123 (DHR 123), and chlorpromazine (CPZ) were obtained from Aladdin (Shanghai, China). IR783 and genistein were purchased from TCI (Shanghai, China). Hydrogen peroxide was purchased from VWR Chemicals. Methyl‐β‐cyclodextrin (M‐β‐CD) was purchased from BLDpharm (Shanghai, China). 2′,7′‐Dichlorodihydrofluorescein diacetate (DCFH‐DA), and EIPA were purchased from MedChemExpress (Shanghai, China). Dimethyl sulfoxide (DMSO) solvent was ordered from Sigma–Aldrich (Darmstadt, Germany). Hoechst 33342, DAPI, Calcein AM, SOSG, and mouse GM‐CSF recombinant protein were obtained from Thermo Fisher (MA, USA). 2,5‐diphenyl‐2‐H‐tetrazolium bromide (MTT) was obtained from J&K Chemical (Beijing, China). Apoptosis assay kit, LDH assay kit, TUNEL assay kit, ATP assay kit, DNA damage kit, and propidium iodide (PI) were purchased from Beyotime (Shanghai, China). Acetonitrile (ACN), methanol (MeOH), and other organic solvents were ordered from Oriental Co., Ltd (Hong Kong, China). Antibodies used in this study were listed in Table S1 (Supporting information).

### Cell Culture

The human triple‐negative breast cancer cell line (MDA‐MB‐231), mouse macrophage‐like cell line (RAW264.7), and mouse breast cancer cell line (4T1) were purchased from ATCC. Cells were cultured in DMEM (Gibco) supplemented with 10% FBS (Gibco) and 1% penicillin/streptomycin (Gibco) at 37 °C with 5% CO_2_.

### Preparation and Characterization of IR/Lap/CUDC NPs

The *β*‐lap (5 mg mL^−1^) and CUDC101 (10 mg mL^−1^) were dissolved in DMSO, respectively. Afterward, 3 µL *β*‐lap and 3 µL CUDC101 were mixed and vortexed thoroughly. Such mixture solution was quickly added into 600 µL IR783 solution using the nanoprecipitation method and stirred for 2 min to fabricate IR/Lap/CUDC NPs. To screen the suitable formulations, gradient drug ratios (0.1, 0.25, 0.375, 0.5, 0.75) or IR783 concentrations (25, 50, 75, 100 µg mL^−1^) were explored. The nanoparticles were further purified by ultracentrifugation (100 kDa MWCO, 1000 g, 5 min) to remove the organic solvent and unloaded drug molecules. The nanoparticles were then sonicated and centrifuged at 3000 g for 3 min to remove large precipitates. The size, polydispersity index (PDI), and Zeta potential were determined by dynamic light scattering (DLS, Malvern Zetasizer). TEM images were captured by FEl Tecnai G2 20 Scanning TEM. The IR/Lap/CUDC NPs were also dissolved in PBS solution for stability test under physiological conditions (37 °C, 48 h). The UV–vis absorption spectra and fluorescent spectra (Ex: 650 nm, Em: 700–850 nm) were determined by SpectraMax M4 (Molecular Devices LLC, San Jose, CA). The H_2_O_2_‐responsiveness of nanoparticles was tested under 10 mm H_2_O_2_ solution incubated for 30 min at 37 °C, and the IR783 decomposition was determined by LC‐MS. The concentration of loaded drug molecules at different drug feeding ratios was determined by high‐performance liquid chromatography (HPLC) to calculate the encapsulation efficiency (weight of loaded drug divided by the weight of fed drug) and loading capacity (weight of loaded drug divided by the weight of nanoparticles).

### Drug Release

The release profile of *β*‐lap and CUDC101 from IR/Lap/CUDC NPs was determined using the dialysis method. 450 µL IR/Lap/CUDC NPs were placed in each dialysis bag (3500 Da MWCO) and dialyzed against 7 mL of PBS buffer with or without H_2_O_2_ at 37 °C. At pre‐determined time points (0.5, 1, 2.5, 4, 6, 8, and 24 h), the whole external PBS solution was replaced with the fresh PBS buffer. The cumulative release percentage over time was measured by HPLC.

### Cytotoxicity Assay

4T1 cells or MDA‐MB‐231 cells (5 × 10^3^) were seeded in 96‐well plates and incubated with gradient concentrations of *β*‐lap, CUDC101, or both for 24 h. Then, 10 µL MTT solution (5 mg mL^−1^) was added into each well and incubated for 3 h. After that, the medium were replaced with 100 µL DMSO, and the absorbance at 490, 570, and 630 nm was determined by SpectraMax M4 to determine the cell viability (normalized to a control group). The synergy scores (HSA model) of *β*‐lap and CUDC101 in 4T1 or MDA‐MB‐231 cells were calculated in the SynergyFinder website according to the cell viability results. To compare the anticancer effect of IR/Lap/CUDC NPs and free drugs, 4T1 cells were treated with different formulations for 24 h. Then, the cell viability was also determined by MTT assay. Besides, the supernatant of treated 4T1 cells in 96‐well plates was collected and LDH release levels were detected following the instructions provided in the kit. To test the cytotoxicity of IR783 dye, the cells were incubated with gradient concentrations of IR783 for 24 h. Then, the cell viability was determined by MTT assay.

### Cellular Uptake

4T1 cells (4 × 10^4^) were seeded in 24‐well plates and cultured overnight. The cells were treated with DMSO, IR783, or equivalent IR/Lap/CUDC NPs for 1 or 4 h. Afterward the cells were washed with PBS, trypsinized, and harvested. The IR783 fluorescence intensity (APC‐Cy7 channel) was determined by flowcytometry (Agilent NovoCyte Quanteon). To investigate the internalization mechanism of IR/Lap/CUDC NPs, 4T1 cells were preincubated with a series of endocytosis inhibitors, including EIPA (20 µm), methyl‐β‐cyclodextrin (M‐β‐CD, 400 µm), chlorpromazine (CPZ, 14 µm), and genistein (74 µm) for 3 h before adding nanoparticles. The IR783 fluorescence intensity was also measured by flowcytometry as described above.

### Live/Dead Cell Staining

4T1 cells (1.5 × 10^5^) were seeded in confocal dishes (SPL100350) and treated with different formulations for 24 h. Then, the cells were incubated with Calcein AM and PI in PBS solution for 20 min at 37 °C. The red and green fluorescence inside cells were imaged by confocal laser scanning microscopy (CLSM, Carl Zeiss LSM880).

### Cell Apoptosis

4T1 cells (3 × 10^4^) were seeded in 24‐well plates and treated with different formulations for 24 h. Afterward the cells were washed with PBS, trypsinized, collected, and stained with an Annexin‐V/PI apoptosis kit according to the instructions. The apoptotic cells were analyzed by flowcytometry (Agilent NovoCyte Quanteon).

### ROS Generation

4T1 cells (4 × 10^4^) were seeded in 24‐well plates and cultured overnight. The cells were incubated with different formulations for 4 h. Next, the cells were washed with PBS and cultured in the fresh medium with a 10 µm DCFH‐DA probe, After 30 min incubation, cells were washed with PBS again and collected for flow cytometric analysis. For CLSM imaging, 4T1 cells (1.5 × 10^5^) were seeded in confocal dishes and treated with different formulations for 4 h. Then, the cells were cultured in the fresh media containing the DCFH‐DA probe for 30 min, washed with PBS, and stained with Hoechst 33342 for ROS detection. For the detection of different types of ROS, the 4T1 cells were stained with hydroxyphenyl fluorescein (HPF), dihydrorhodamine 123 (DHR 123), or SOSG for 30 min, respectively. Then, the cells were washed with PBS and stained with Hoechst 33342 for CLSM imaging.

### NQO1‐Dependent ROS Generation and Cytotoxicity

To explore the mechanism of ROS generation from IR/Lap/CUDC NPs, 4T1 cells (4 × 10^4^) were seeded in 24‐well plates and treated with different formulations for 4 h with or without dicoumarol (DIC, 70 µm) pretreatment. The ROS level inside 4T1 cells was measured by flowcytometry and observed by CLSM imaging. Besides, the cell viability and apoptosis level of 4T1 cells treated with *β*‐lap, *β*‐lap + CUDC101, or IR/Lap/CUDC NPs for 24 h with or without DIC pretreatment were determined by MTT assay and apoptosis assay, respectively. To test the ROS‐responsiveness, 4T1 cells were incubated with IR/Lap/CUDC NPs with or without DIC pretreatment, and the intracellular IR783 fluorescence at indicated time points (0.5, 1, 2, 4 h) was measured by flowcytometry.

### DNA Damage

4T1 cells (1.5 × 10^5^) were seeded in confocal dishes and treated with different formulations for 24 h. Then, the cells were washed with PBS three times, fixed with 4% PFA solution, stained following the instructions provided in the kit, and imaged by CLSM.

### Western Blot Analysis

4T1 cells (1.5 × 10^5^) were seeded in six‐well plates and incubated with different formulations for 24 h. Then, the cells were washed with PBS buffer and collected using RIPA buffer (supplemented with protease and phosphatase inhibitor cocktail). Protein concentration was determined by BCA kit (Thermo Fisher 23225) and protein expression levels (Ac‐α‐Tubulin, Ac‐Histone H3, and GAPDH) were analyzed by polyacrylamide gel electrophoresis. The quantitative results were analyzed by Image Lab software.

### Lateral and Vertical Migration Assay

For lateral migration assay, 4T1 cells (1.5 × 10^5^) were seeded in 24‐well plates and cultured overnight. The 200 µL pipette tip was used to scratch a straight line along the diameter of each well on the cell monolayer. After washing with PBS, 4T1 cells were treated with different formulations for 24 h. The width of each scratch at 0 or 24 h was observed by microscope to calculate the migration level (normalized to a control group). For vertical migration assay, 4T1 cells (2 × 10^4^) were seeded on the upper chamber of the cell culture insert (Costar 3464) and treated with different formulations in 200 µL media without FBS. The lower chamber was filled with 600 µL fresh media with 10% FBS. After 24 h, the cells were fixed with 4% PFA solution and stained with 0.1% crystal violet solution. The migrated cells were imaged by the microscope and counted by ImageJ software to calculate the migration level (normalized to a control group).

### Transcriptome Sequencing and Analysis

Normal 4T1 cells and IR/Lap/CUDC NPs treated 4T1 cells were collected and total RNA was extracted and sent to Genewiz company (Suzhou, China) to construct library and conduct high throughput sequencing. DEGs analysis was performed and displayed as a volcano plot. The heatmap plot was generated using the pheatmap package under the R environment. GO enrichment and KEGG enrichment analysis were performed as well using R.

### Immunogenic Cell Death

4T1 cells (1.5 × 10^5^) were seeded in confocal dishes and treated with different formulations for 24 h. Then, the cells were washed with PBS and fixed with a 4% PFA solution. Next, the cells were washed with PBS, permeabilized with 0.3% Triton X‐100, and blocked in 3% BSA in PBS for 1 h at room temperature. After incubating with primary antibodies (Anti‐Calreticulin (CRT) or Anti‐HMGB1 antibody), secondary antibody (FITC‐conjugated Goat Anti‐Rabbit IgG H&L), and DAPI (Thermo Fisher D1306) according to the instruction, the cells were mounted with SlowFade Diamond Antifade Mountant (Thermo Fisher S36963). The immunofluorescence was observed by CLSM and the quantitative results were analyzed by ImageJ software. To measure the ATP level, 4T1 cells (3 × 10^4^) were seeded in 24‐well plates and treated with different formulations for 24 h. The intracellular ATP level was detected following the instructions provided in the kit.

### Activation of BMDCs In Vitro

Mouse bone marrow‐derived dendritic cells (BMDCs) were collected from the femur and tibia of healthy C57BL/6J mice, and incubated with GM‐CSF for 6 days. 4T1 cells (3 × 10^4^) were seeded in 24‐well plates and treated with different formulations for 24 h. Then cell culture supernatants of different groups were added to BMDCs seeded in a 24‐well. After 24 h, BMDCs were collected and stained with anti‐CD11c (FITC), anti‐CD80 (PE), and anti‐CD86 (BV785) antibodies according to the instructions. The BMDC maturation level was analyzed by flow cytometry (Agilent NovoCyte Quanteon).

### RNA Extraction and Reverse Transcription‐PCR

RAW264.7 cells (3 × 10^4^) were seeded in 12‐well plates and cultured overnight. The macrophages were first incubated in the complete medium with 40 ng mL^−1^ mouse IL‐4 (Abcam ab259406) and IL‐13 (Abcam ab270080) for 48 h. Then the induced macrophages were treated with different formulations for 24 h. After washing with PBS, total RNA was extracted using an RNA extraction kit (Takara 9108) according to the instructions. The RNA was reverse‐transcribed using the PrimeScript RT reagent kit (Takara RR037A) for cDNA production by a thermal cycler (Applied Biosystems, Thermo Fisher). The amplification of pro‐inflammatory genes (TNF‐α, IL‐1β) and a control gene (GAPDH) was analyzed by quantitative polymerase chain reaction (qPCR) according to the instruction (Takara RR039A) via LightCycler 480 system (Roche diagnostics). The sequences of relevant primers were as follows: TNF‐α, forward 5′‐CCCTTTACTCTGACCCCTTTATTGT‐3′, reverse 5′‐TGTCCCAGCATCTTGTGTTTCT‐3′. IL‐1β, forward 5′‐TCCAGGATGAGGACATGAGCAC‐3′, reverse 5′‐GAACGTCACACACCAGCAGGTTA‐3′. GAPDH, forward 5′‐TGTGTCCGTCGTGGATCTGA‐3′, reverse 5′‐TTGCTGTTGAAGTCGCAGGAG‐3′.

### Flowcytometric Analysis of CD80

RAW264.7 cells (3 × 10^4^) were seeded in 12‐well plates and cultured in the complete medium with IL‐4 and IL‐13. After different drug treatments, the macrophages were stained with anti‐CD80 (APC) antibodies for flowcytometric analysis.

### Animal Studies

BALB/c mice (4–6 weeks) were obtained from the Centre for Comparative Medicine Research (Li Ka Shing Faculty of Medicine, The University of Hong Kong). All animals received care and experiments in accordance with the guidelines and protocol approved by the Committee on the Use of Live Animals in Teaching and Research (CULTAR) at Li Ka Shing Faculty of Medicine (CULTAR No. 22–172). Animals were maintained at the CA‐DMB (Centre for Comparative Medicine Research). For subcutaneous 4T1 tumor‐bearing BALB/c mouse model, 4T1 cells (8 × 10^5^) in 100 µL PBS solution with Matrigel (BD Biosciences) were subcutaneously implanted in the right flank of the mice. The treatment procedures were initiated when the tumor volume reached ≈100 mm^3^. Tumor volume (mm^3^) was calculated as 0.5^*^length^*^width^2^.

### In Vivo Biodistribution

The 4T1 tumor‐bearing mice were intravenously injected with 200 µL free IR783 (0.8 mg kg^−1^) or IR/Lap/CUDC NPs via the tail vein. The IR783 fluorescence intensity was observed at indicated time points (1, 3, 6, 9, 24 h). The mice were euthanized 24 h after injection and the heart, liver, spleen, lung, kidney, and tumor tissues were collected for fluorescence imaging conducted by IVIS Lumina X5 (Cy7 channel). The quantitative results of ex vivo fluorescence at the endpoint and tumor tissue fluorescence at different time points were analyzed by Living Image software.

### AntiTumor Growth Study

The 4T1 tumor‐bearing BALB/c mice were randomly divided into five groups when the tumor size reached ≈100 mm^3^. Different groups of mice were treated with different formulations via tail vein intravenous injection at Day 0, 2, 4, 6, 8: 1) PBS; 2) *β*‐lap; 3) CUDC101; 4) *β*‐lap and CUDC101; 5) IR/Lap/CUDC NPs. The dose for each formulation was set as 2.5 mg kg^−1^ *β*‐lap and 3.5 mg kg^−1^ CUDC101 equivalent. The tumor size and body weight of the mice were measured in a two‐day interval. At the end of the experiment (Day 10), all mice were euthanized to harvest tumor tissues that were weighed and photographed. Besides, the tumor‐draining lymph nodes (TDLNs) under the armpit and main organs were also collected for further analysis. The tumor tissues were further made into paraffin sections and sliced for H&E staining, TUNEL assay, and CRT immunofluorescence analysis.

### Biocompatibility Study

Paraffin‐embedded organ sections were prepared and the morphology of cells was observed after H&E staining using microscope. Alanine aminotransferase (ALT) and Aspartate transaminase (AST) activities in serum samples from treated mice were also detected by ALT and AST detection kits (Solarbio BC1550, BC1560) following the instructions provided.

### Distant Tumor Inhibition Study

To establish a bilateral 4T1 tumor‐bearing BALB/c mouse model, 4T1 cells (3.5 × 10^5^) were first subcutaneously injected into the right flank of the mice as primary tumors (Day ‐7). Afterward, 4T1 cells (1.2 × 10^5^) were subcutaneously implanted into the left flank of the mice as distant tumors (Day 0). The mice were randomly divided into three groups and treated with different formulations daily via tail vein intravenous injection from Day 0 to Day 4: 1) PBS; 2) *β*‐lap and CUDC101; 3) IR/Lap/CUDC NPs. Primary and distant tumor sizes as well as body weight were measured every two days. After 16‐day therapeutic monitoring, the mice were euthanized to harvest primary and distant tumors that were weighed and photographed. Besides, spleens were also collected for further analysis.

### In Vivo Immune Cells Analysis

To explore dendritic cell maturation, TDLNs collected in antitumor growth study were first digested with 1 mg mL^−1^ type IV collagenase and 0.2 mg mL^−1^ DNase I in the RPMI complete medium for 40 min at 37 °C. Then, the mixtures were filtered through the 70 µm nylon cell strainer (SPL 93070) to obtain single cell suspension. The cells were washed using PBS and centrifuged at 400 g for 3 min, which was repeated three times. Zombie Yellow Fixable Viability Kit (Biolegend 423103) was used for Live/Dead staining. Afterward, the cells were incubated with anti‐CD45, anti‐I‐A/I‐E, anti‐CD11c, anti‐CD80, and anti‐CD86 antibodies on ice for 40 min in the dark. At last, the cells were washed with cell staining buffer for flowcytometric analysis. To analyze the effector memory T cells, spleens harvested in distant tumor inhibition study were made into single cell suspensions and stained with Zombie Yellow, anti‐CD3, anti‐CD8, anti‐CD44, and anti‐CD62L antibodies for flowcytometric analysis. For tumor‐infiltrating T cell analysis, primary and distant tumors collected in distant tumor inhibition study were made into single cell suspensions and incubated with Zombie Yellow, anti‐CD45, anti‐CD3, anti‐CD4, anti‐CD8 antibodies for flowcytometric analysis. To analyze tumor‐associated macrophages, single‐cell suspensions from primary tumor tissues harvested in distant tumor inhibition study were washed using PBS, centrifuged at 400 g for 3 min, and incubated with Zombie Yellow, anti‐CD45, anti‐F4/80, anti‐CD80, and anti‐CD206 antibodies for flowcytometric analysis.

### Statistical Analysis

All experiments were conducted three times or more independently (*n* ≥ 3). Data were presented as the mean ± standard deviation (SD). GraphPad Prism 8.3.0 software (GraphPad Software, Inc.) was used for statistical data analysis. To compare the differences between the two groups, a two‐tailed unpaired Student's *t*‐test was adopted. To compare the differences between multiple‐group means, a one‐way analysis of variance (ANOVA) was used. P value less than 0.05 was considered statistically significant.

## Conflict of Interest

The authors declare no conflict of interest.

## Supporting information



Supporting Information

## Data Availability

The data that support the findings of this study are available from the corresponding author upon reasonable request.
