# Supplementary material for: IR783‐Stabilized Nanodrugs Enhance Anticancer Immune Response by Synergizing Oxidation Therapy and Epigenetic Modulation
Source: Adv Sci (Weinh). 2025 Mar 27;12(21):2415684. doi: 10.1002/advs.202415684 (PMC12140360; doi:10.1002/advs.202415684)
Supplement: Supplementary file 1 — Supporting Information [file ADVS-12-2415684-s001.docx]

Supporting Information

IR783-stabilized nanodrugs enhance anti-cancer immune response by synergizing oxidation therapy and epigenetic modulation

Jinzhao Liu, Meicen Wu, Qingyang Lyu, Chang Yang, Ni Fan, Kang Chen, Weiping Wang*

Jinzhao Liu, Meicen Wu, Qingyang Lyu, Chang Yang, Ni Fan, Weiping Wang

State Key Laboratory of Pharmaceutical Biotechnology, The University of Hong Kong, Hong Kong, China

Department of Pharmacology and Pharmacy, Li Ka Shing Faculty of Medicine, The University of Hong Kong, Hong Kong, China

Dr. Li Dak-Sum Research Centre, The University of Hong Kong, Hong Kong, China

E-mail: wangwp@hku.hk

Kang Chen

State Key Laboratory of Pharmaceutical Biotechnology, The University of Hong Kong, Hong Kong, China

Dr. Li Dak-Sum Research Centre, The University of Hong Kong, Hong Kong, China

Department of Medicine, Li Ka Shing Faculty of Medicine, The University of Hong Kong, Hong Kong, China

| **Name** | **Cat #** | **Fluorophore** | **Company** |
| --- | --- | --- | --- |
| Anti-Ac-α-Tubulin (Lys40) antibody | 66200-1-lg | N/A | Proteintech |
| Anti-Ac-Histone H3 (Lys9) antibody | 29133-1-AP | N/A | Proteintech |
| Anti-GAPDH antibody | 60004-1-lg | N/A | Proteintech |
| Anti-rabbit IgG H&L (HRP) | ab6721 | N/A | Abcam |
| Anti-HMGB1 antibody | A19529 | N/A | ABclonal |
| Anti-Calreticulin antibody | A1066 | N/A | ABclonal |
| Goat Anti-Rabbit IgG H&L | ab6717 | FITC | Abcam |
| Anti-CD11c antibody | 117305 | FITC | Biolegend |
| Anti-CD80 antibody | 104707 | PE | Biolegend |
| Anti-CD86 antibody | 105043 | BV785 | Biolegend |
| Anti-CD80 antibody | 104714 | APC | Biolegend |
| Anti-CD206 antibody | 141706 | PE | Biolegend |
| Anti-CD45 antibody | 157214 | FITC | Biolegend |
| Anti-I-A/I-E antibody | 107623 | PerCP | Biolegend |
| Anti-CD11c antibody | 117321 | Pacific Blue | Biolegend |
| Anti-CD86 antibody | 105014 | PE-Cy7 | Biolegend |
| Anti-CD3 antibody | 100221 | APC-Cy7 | Biolegend |
| Anti-CD4 antibody | 100427 | Pacific Blue | Biolegend |
| Anti-CD8 antibody | 100733 | PerCP-Cy5.5 | Biolegend |
| Anti-CD44 antibody | 103009 | PE-Cy5 | Biolegend |
| Anti-CD62L antibody | 161211 | FITC | Biolegend |
| Anti-F4/80 antibody | 123125 | PerCP | Biolegend |

**Table S1:** The antibodies used in this work for Western blot, Immunofluorescence, and Flow cytometry experiments.


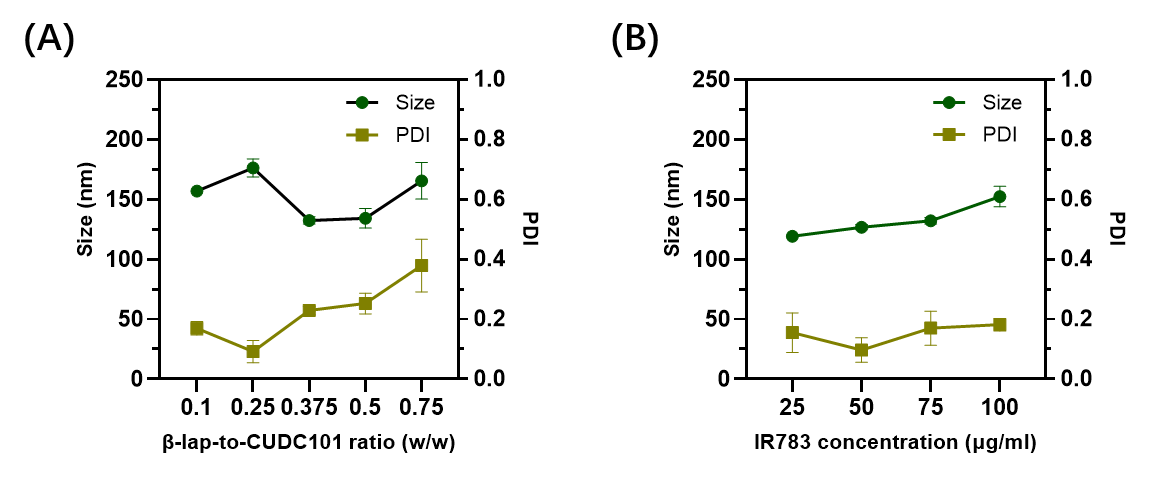


**Figure S1:** (A) Hydrodynamic sizes and PDI values of IR/Lap/CUDC NPs at different drug feeding ratios in 50 μg/mL IR783 solution. (B) Hydrodynamic sizes and PDI values of IR/Lap/CUDC NPs at the *β*-lap-to-CUDC101 feeding ratio of 0.5 with different IR783 concentration.


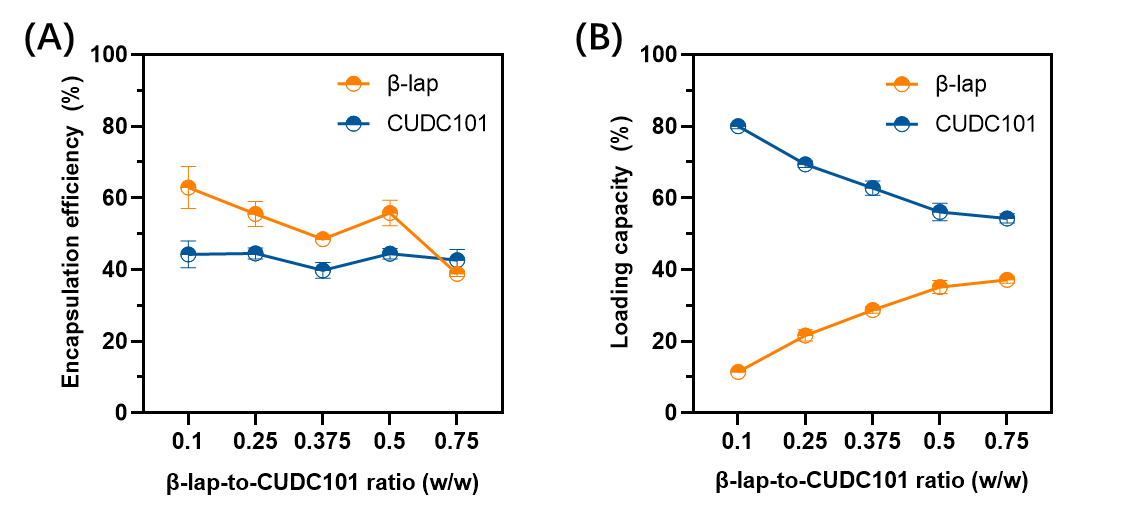


**Figure S2:** Encapsulation efficiency (A) and loading capacity (B) of *β*-lap and CUDC101 at different drug feeding ratios.


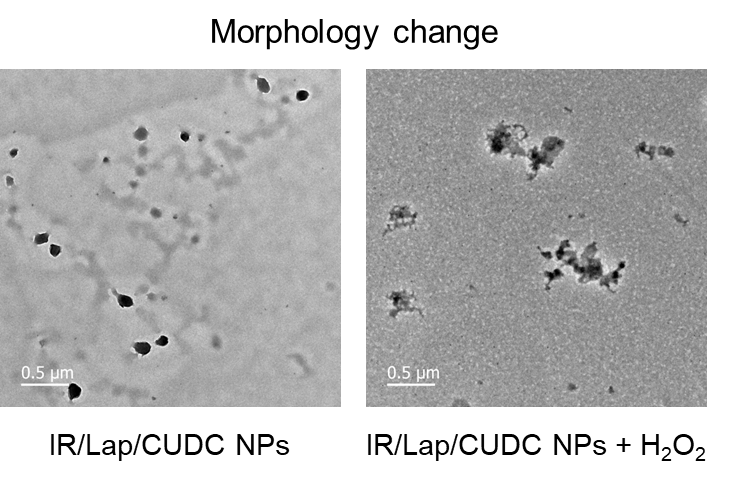


**Figure S3:** Morphology changes of IR/Lap/CUDC NPs with or without H_2_O_2_ under transmission electron microscopy (TEM) imaging (Scale bar: 500 nm).


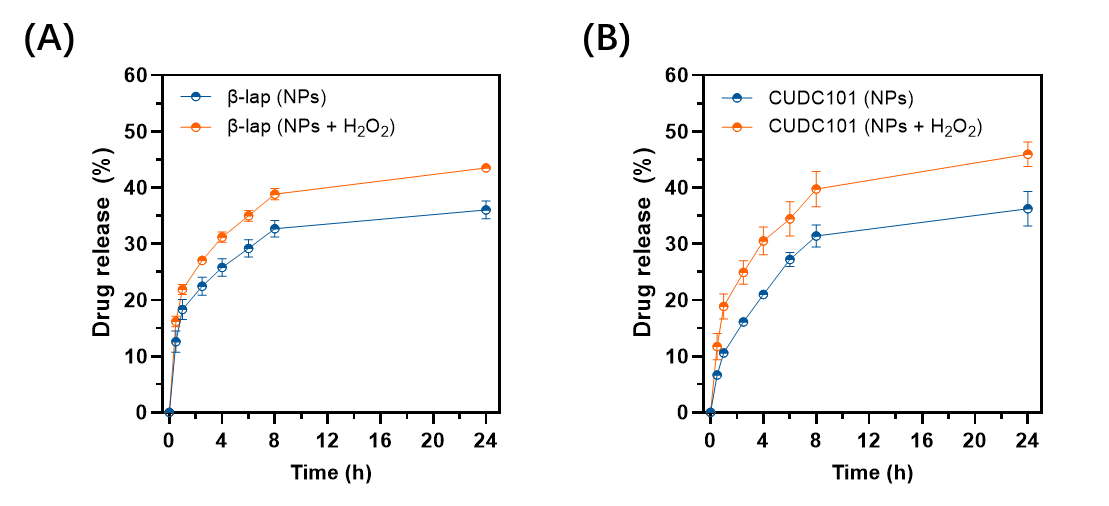


**Figure S4:** (A) *β*-lap drug release profiles of IR/Lap/CUDC NPs with or without H_2_O_2_. (B) CUDC101 drug release profiles of IR/Lap/CUDC NPs with or without H_2_O_2_.


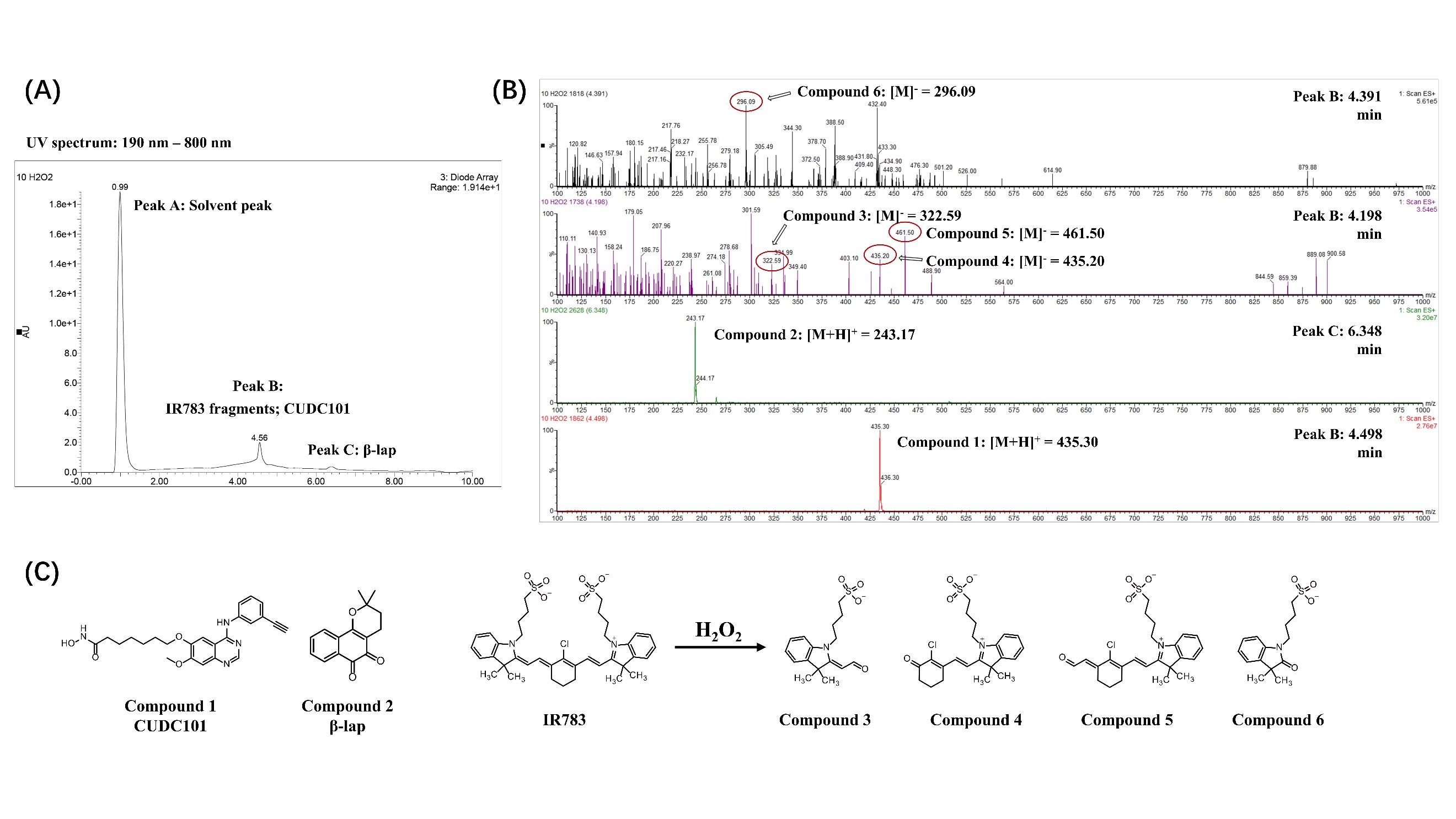


**Figure S5:** (A) Liquid chromatography (LC) trace of IR/Lap/CUDC NPs incubated with H_2_O_2_ (Absorption range: 190 - 800 nm). (B) LC-MS spectra of IR/Lap/CUDC NPs incubated with H_2_O_2_. (C) Schematic illustration of H_2_O_2_-responsive IR783 decomposition.


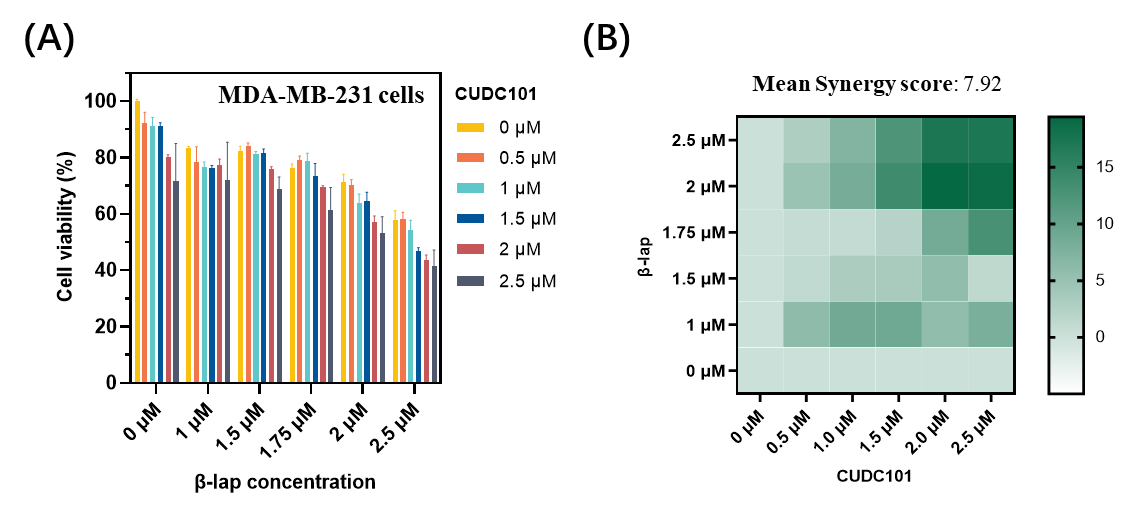


**Figure S6:** (A) Cell viability of MDA-MB-231 cells treated with gradient concentrations of free *β*-lap and CUDC101. (B) Synergy score of free *β*-lap and CUDC101 in MDA-MB-231 cells at gradient concentrations calculated by HSA model in SynergyFinder.

**Figure S7:** Cell viability of 4T1 cells treated with gradient concentrations of IR783.


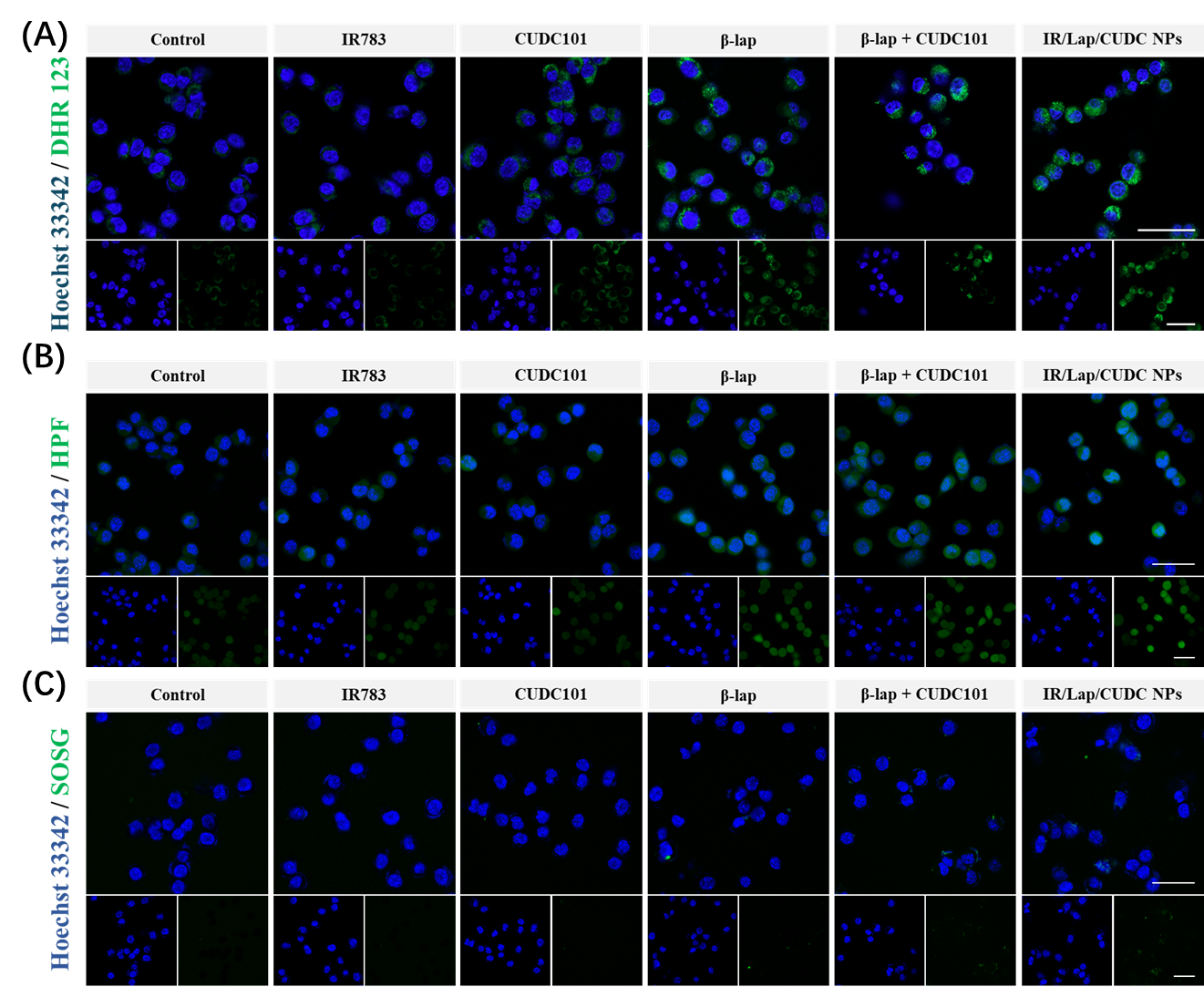


**Figure S8:** Representative CLSM images of superoxide anion ·O_2_^-^ (A), hydroxyl radical ·OH and peroxynitrite anion ONOO^-^ (B), and singlet oxygen ^1^O_2_ (C) in 4T1 cells after different treatments (Scale bar: 50 μm, Hoechst 33342: nucleus in blue, DHR 123: ·O_2_^-^ in green, HPF: ·OH and ONOO^-^ in green, SOSG: ^1^O_2_ in green).


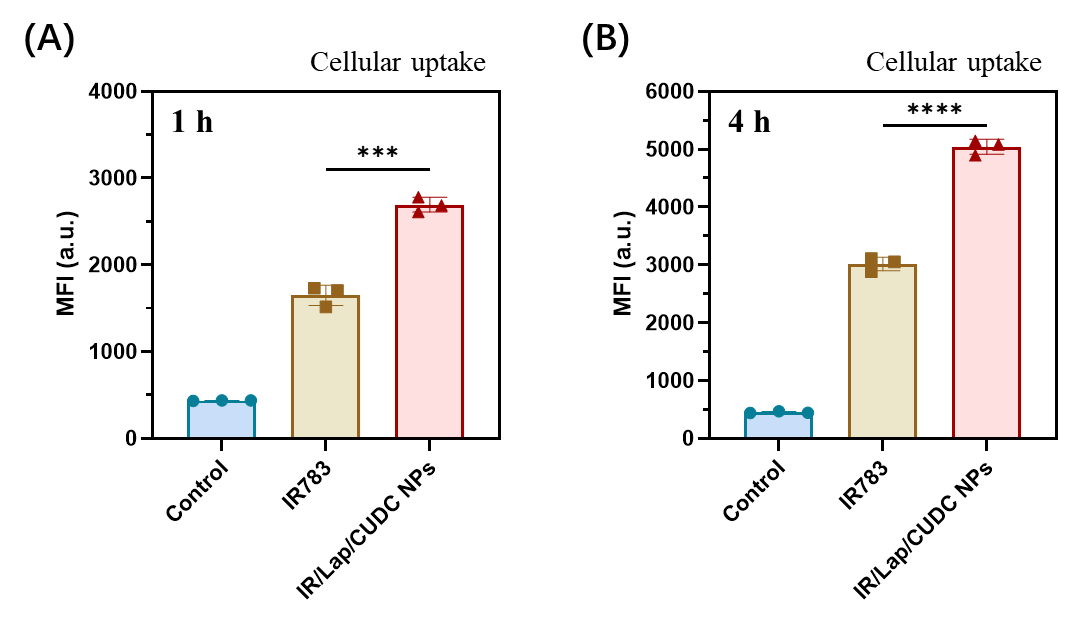


**Figure S9:** Flowcytometric analysis of cellular uptake behavior in 4T1 cells treated with free IR783 or IR/Lap/CUDC NPs for 1 h (A) or 4 h (B) incubation.


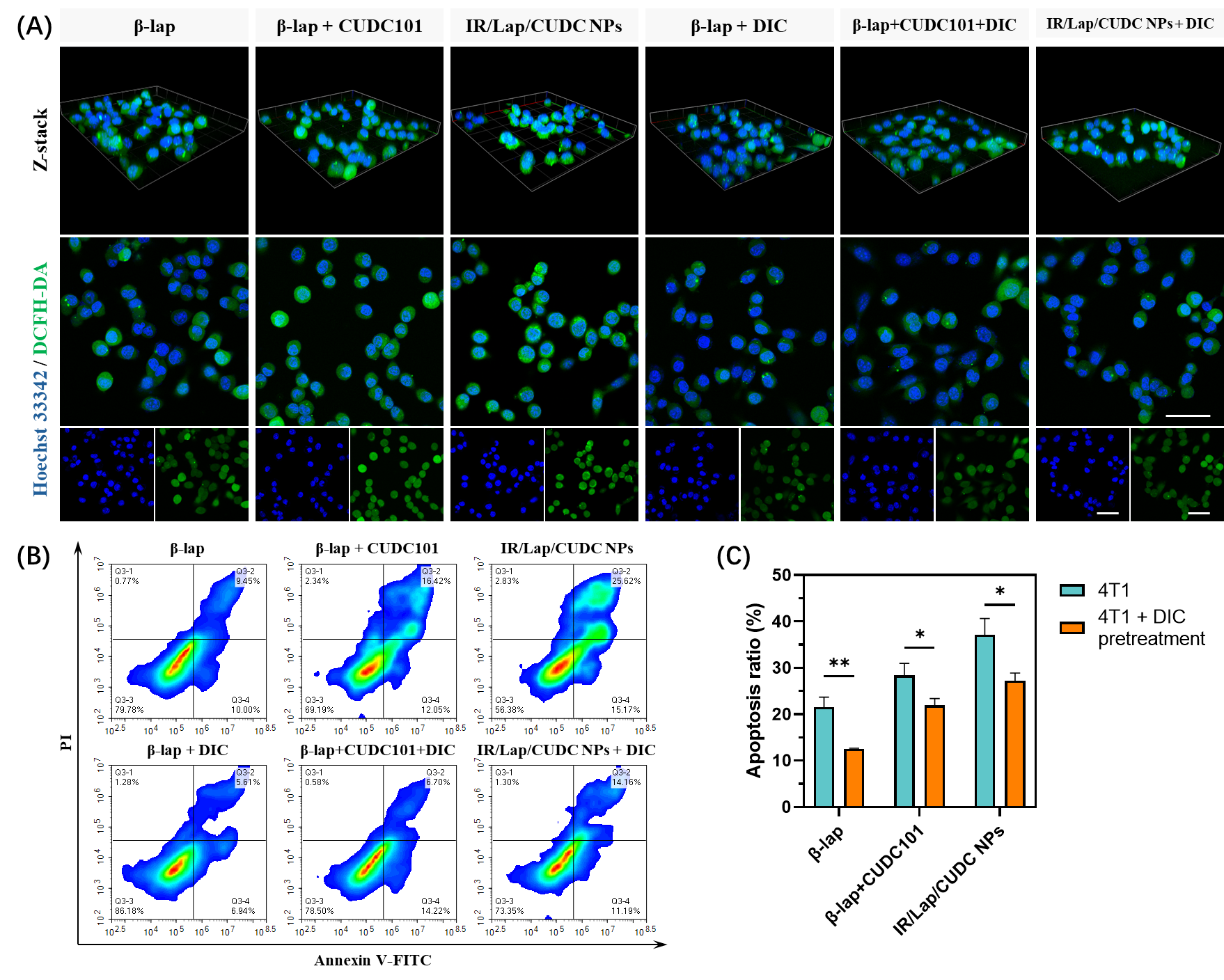


**Figure S10:** (A) Representative CLSM images of ROS in 4T1 cells after different treatments with or without dicoumarol (DIC) (Scale bar: 50 μm). (B) Apoptosis level of 4T1 cells after different treatments with or without DIC. (C) Quantitative analysis of apoptosis level after different treatments with or without DIC.

**Figure S11:** Flowcytometric analysis of ROS generation in 4T1 cells treated with *β*-lap and different concentrations of IR783.


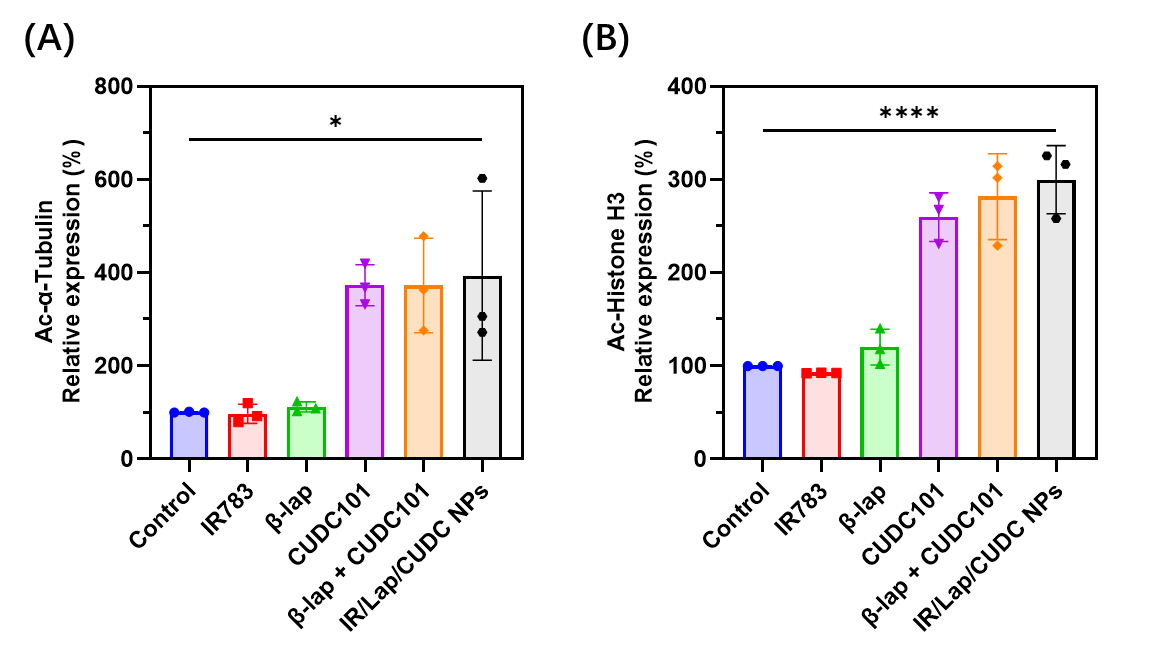


**Figure S12:** Quantitative western blot analysis of acetylated α-tubulin (A) and acetylated histone H3 (B) relative expression levels in 4T1 cells after different treatments.

**Figure S13:** Quantitative analysis of γ-H2AX confocal imaging assay.


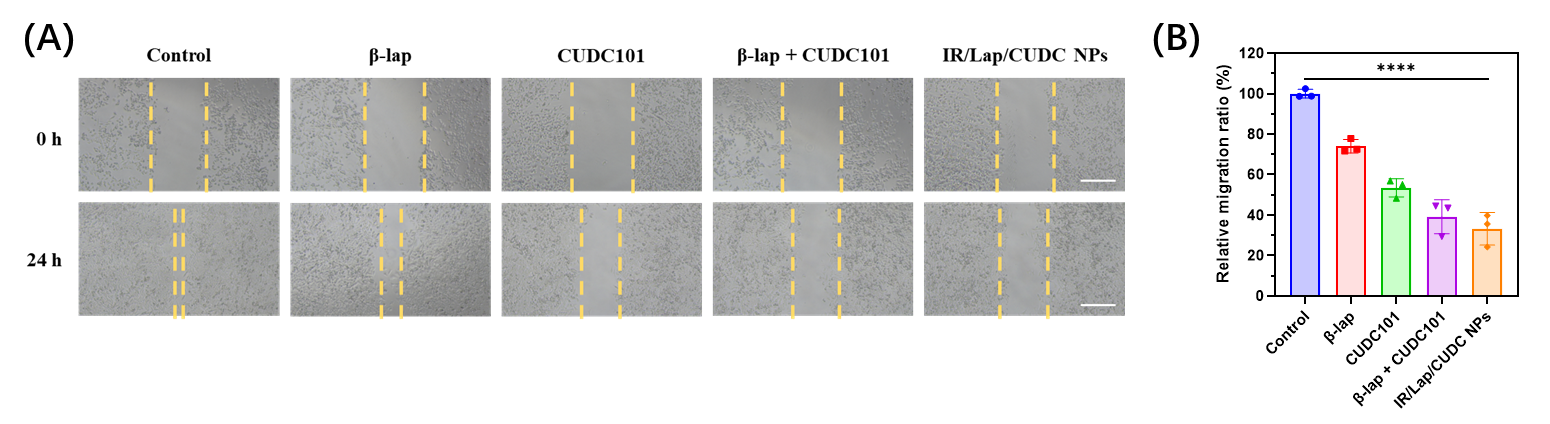


**Figure S14:** (A) Lateral migration of 4T1 cells after different treatments for 24 h (Scale bar: 0.5 mm). (B) Quantitative analysis of lateral migration assay.


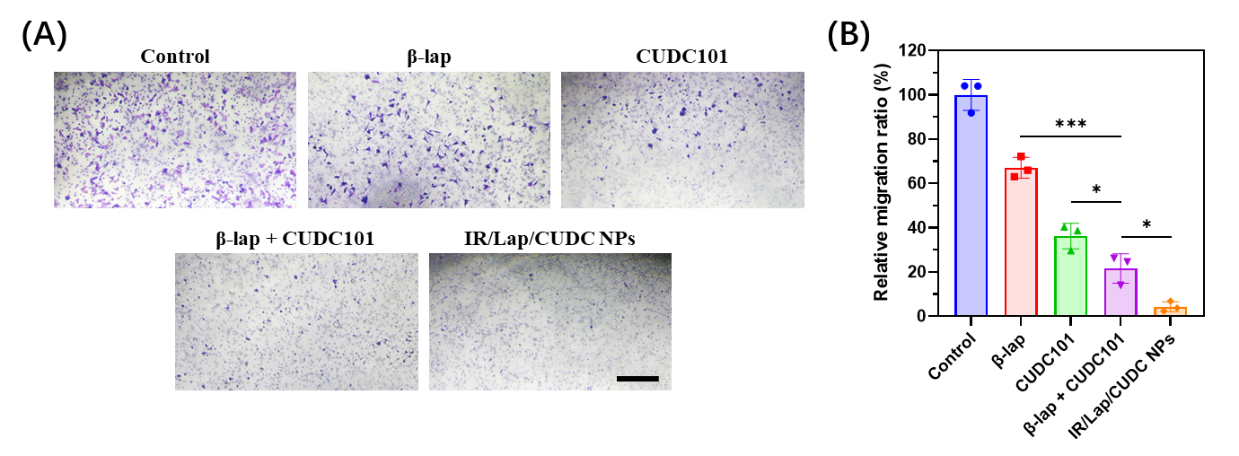


**Figure S15:** (A) Vertical migration of 4T1 cells after different treatments for 24 h (Scale bar: 0.5 mm). (B) Quantitative analysis of vertical migration assay.


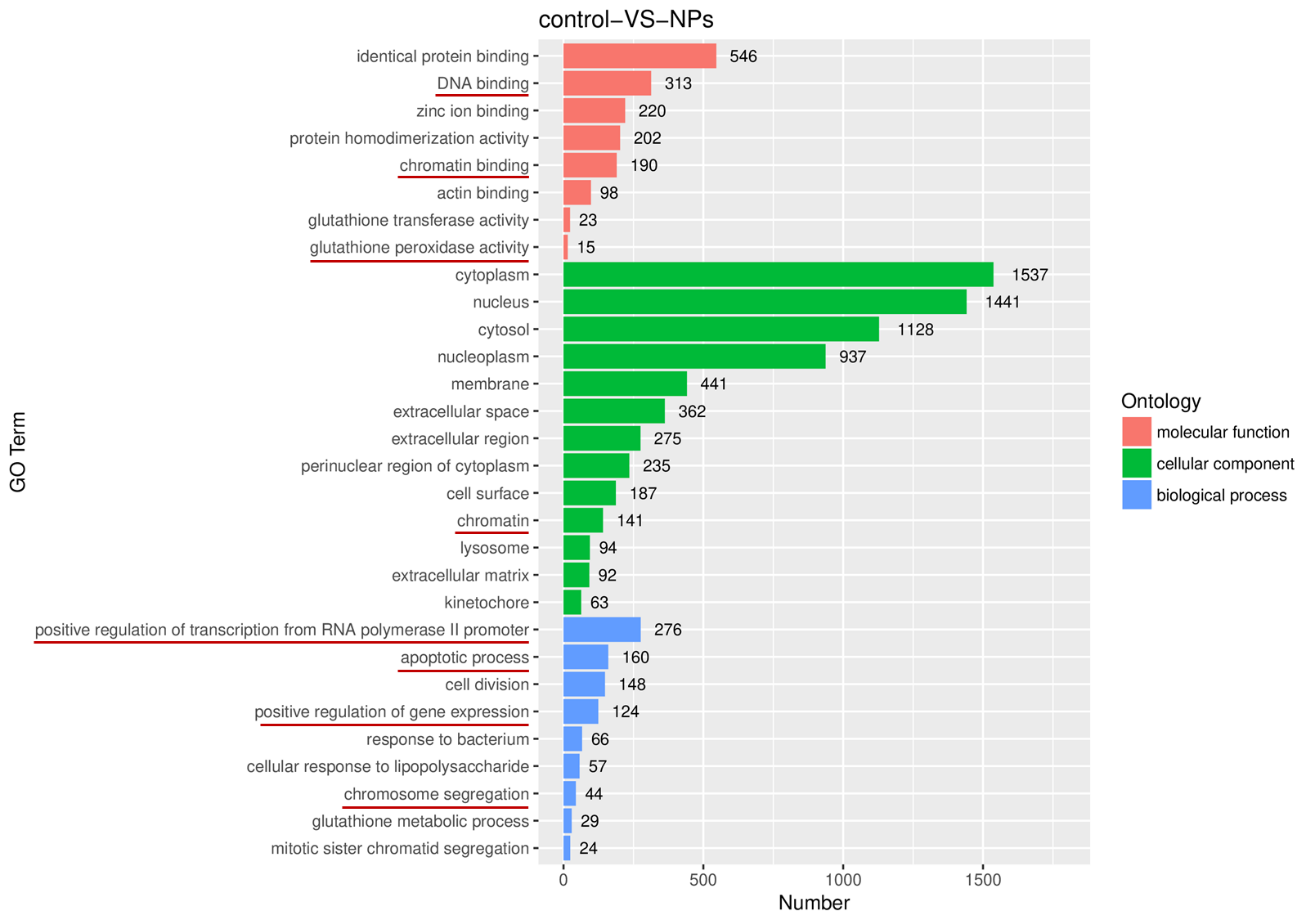


**Figure S16:** GO enrichment analysis of DEGs between normal 4T1 cells and IR/Lap/CUDC NPs treated 4T1 cells.


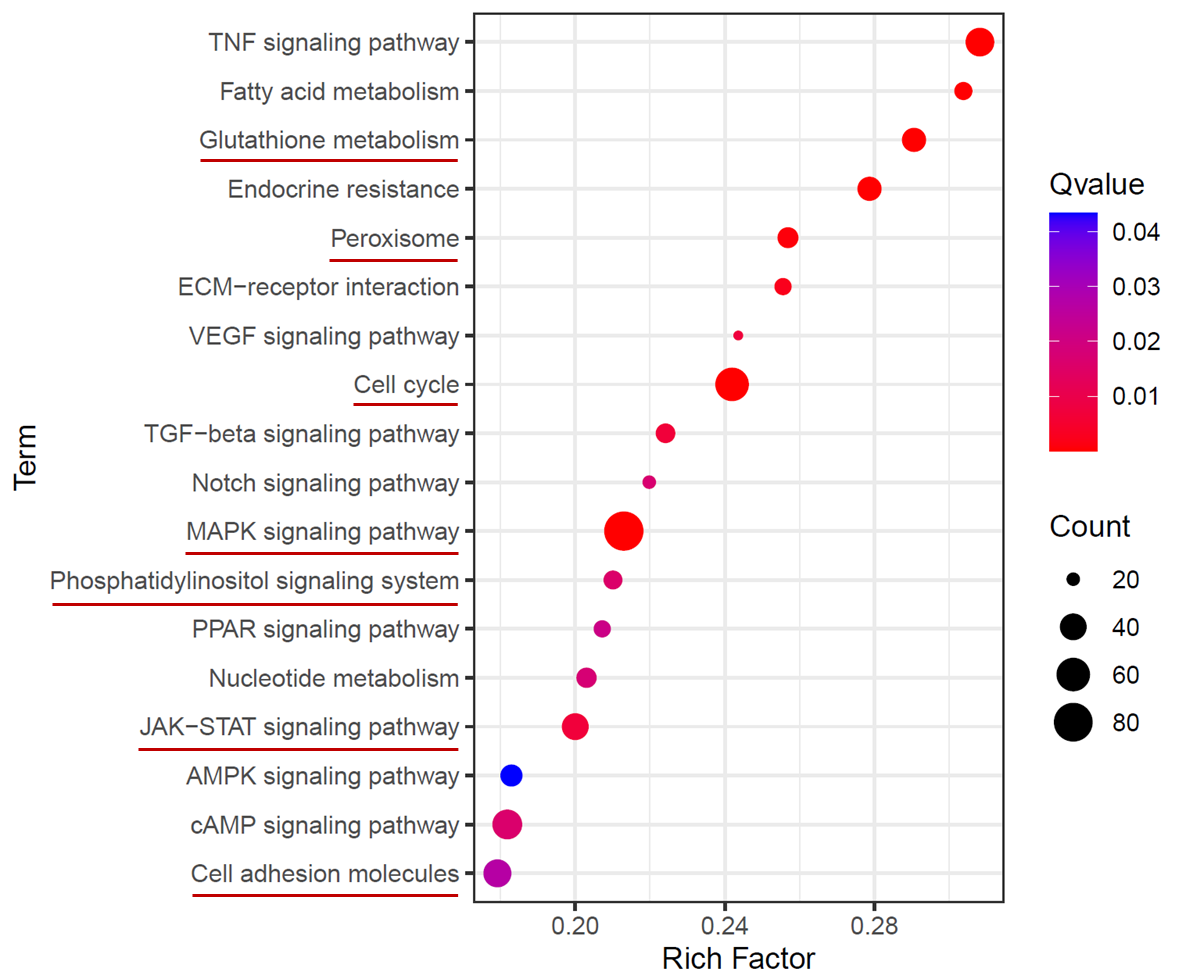


**Figure S17:** KEGG enrichment analysis of DEGs between normal 4T1 cells and IR/Lap/CUDC NPs treated 4T1 cells (Q value < 0.05).


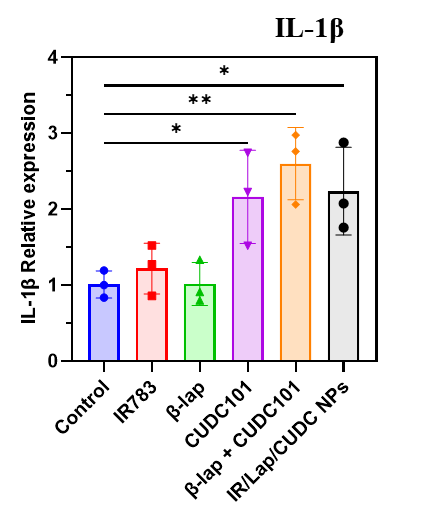


**Figure S18:** mRNA expression of IL-1β in RAW264.7 cells after different treatments.


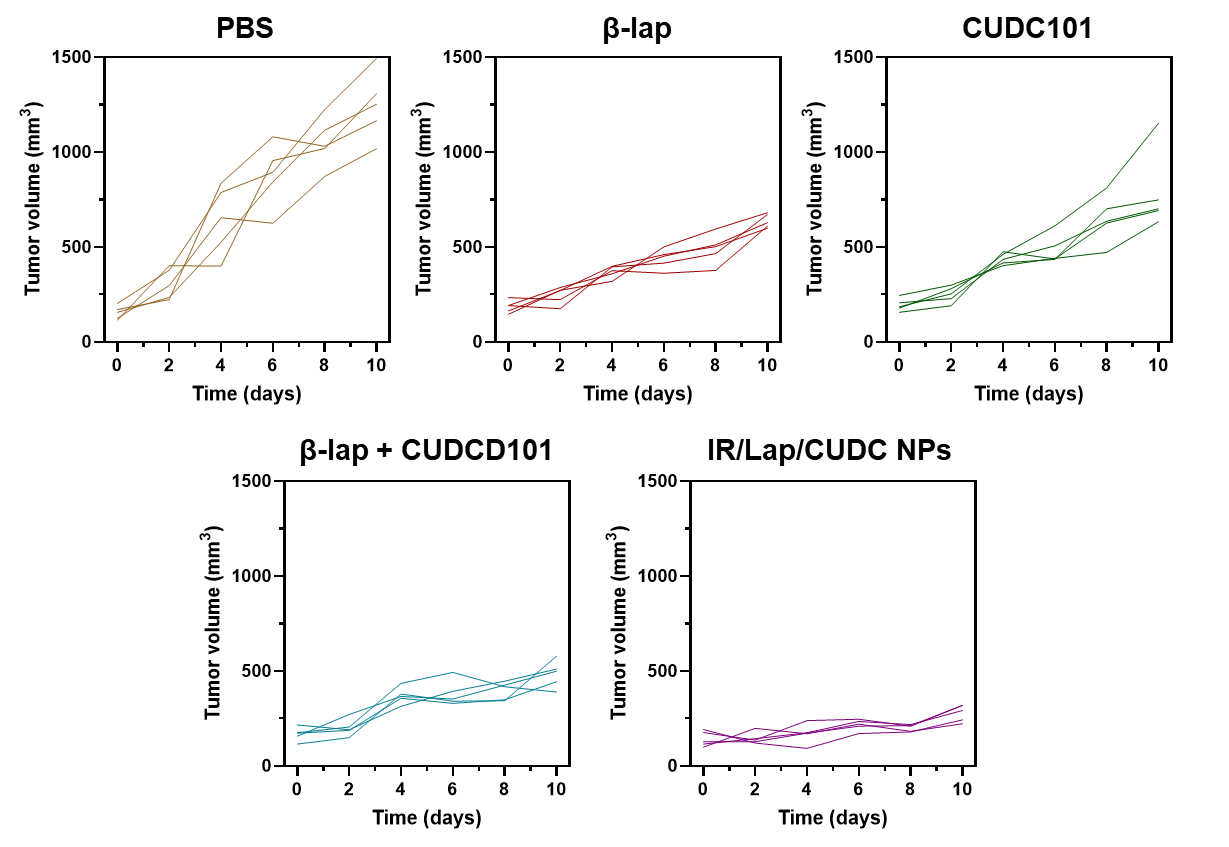


**Figure S19:** Individual tumor volume growth profiles of tumor-bearing mice with different treatments in the anti-tumor growth study.


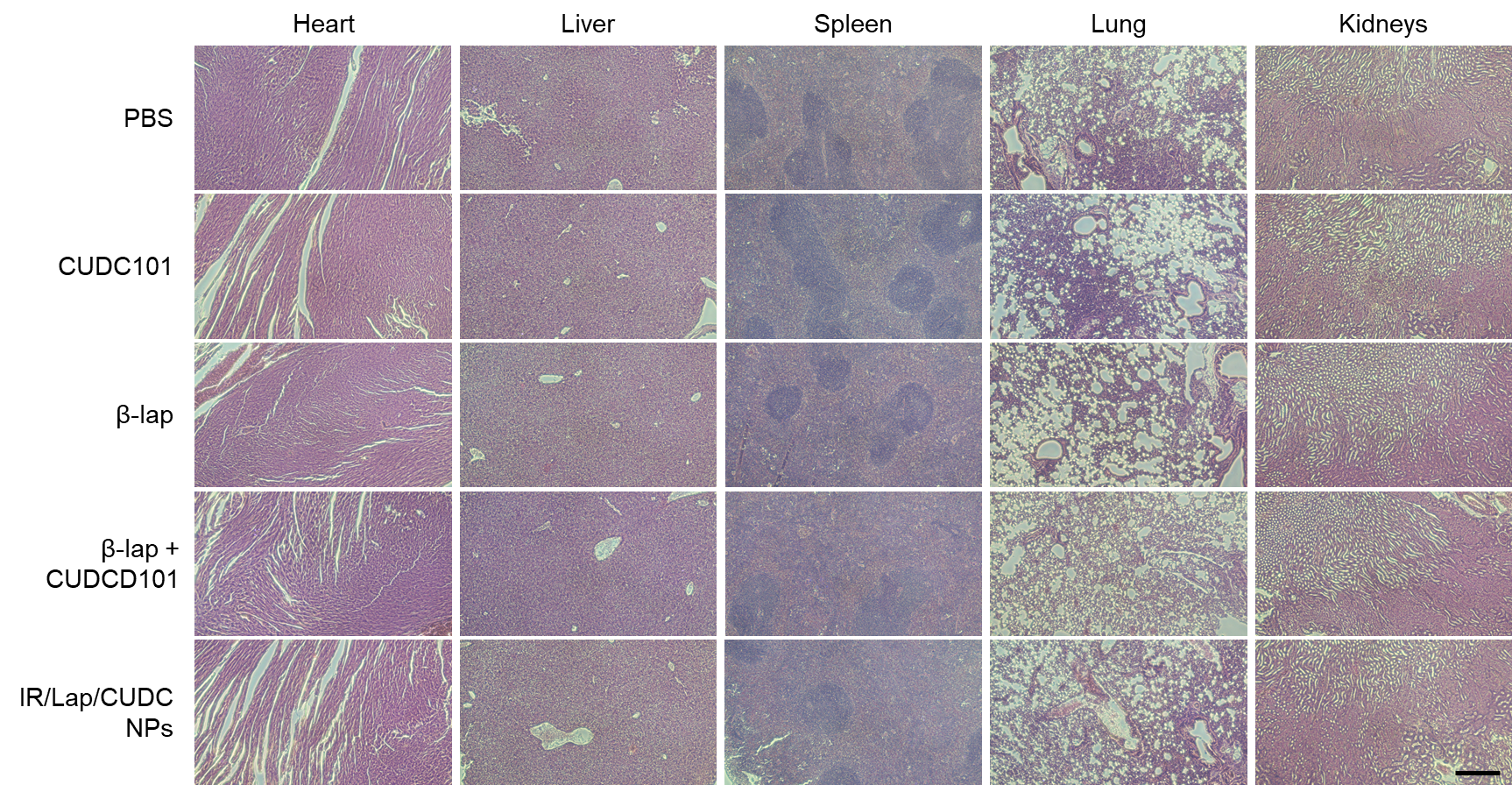


**Figure S20:** H&E staining assessment of major organs (heart, liver, spleen, lung, and kidney) from the 4T1 tumor-bearing mice after different treatments (Scale bar: 0.5 mm).


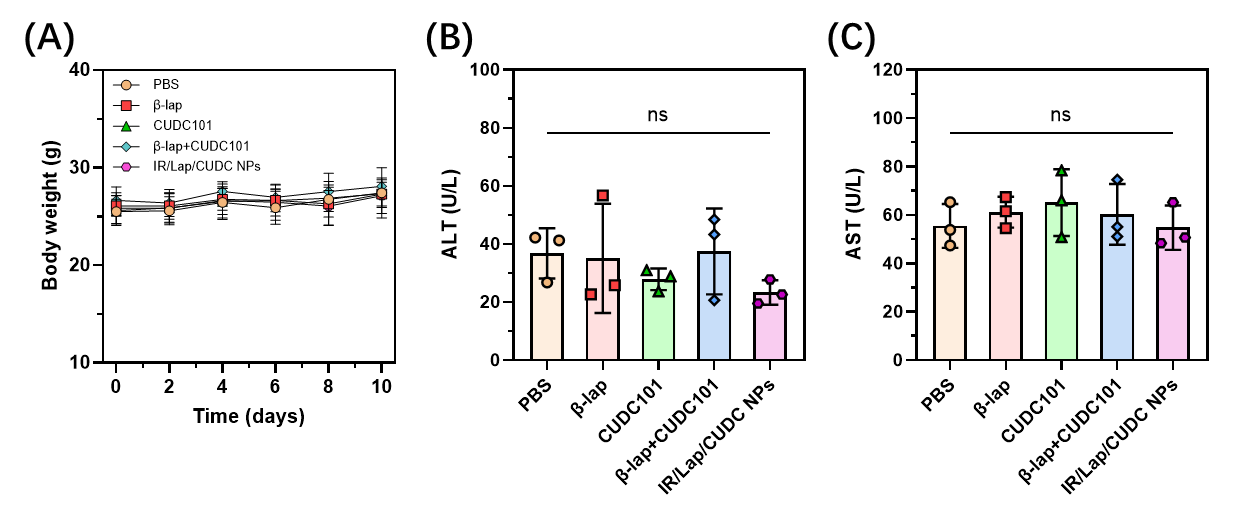


**Figure S21:** (A) Body weight change of the 4T1 tumor-bearing mice receiving different treatments during 10-day treatment in the anti-tumor growth study. (B) ALT activity analysis of serum samples from mice treated with different formulations. (C) AST activity analysis of serum samples from mice treated with different formulations.


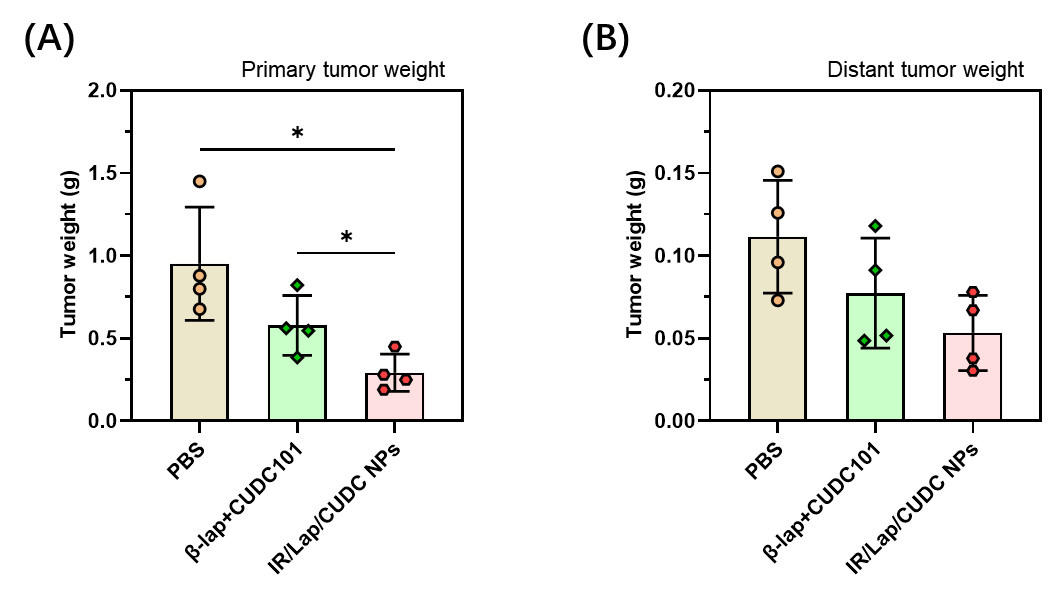


**Figure S22:** (A) Primary tumor weight of different treatment groups on day 16 in bilateral 4T1 tumor-bearing BALB/c mice model. (B) Distant tumor weight of different treatment groups on day 16.


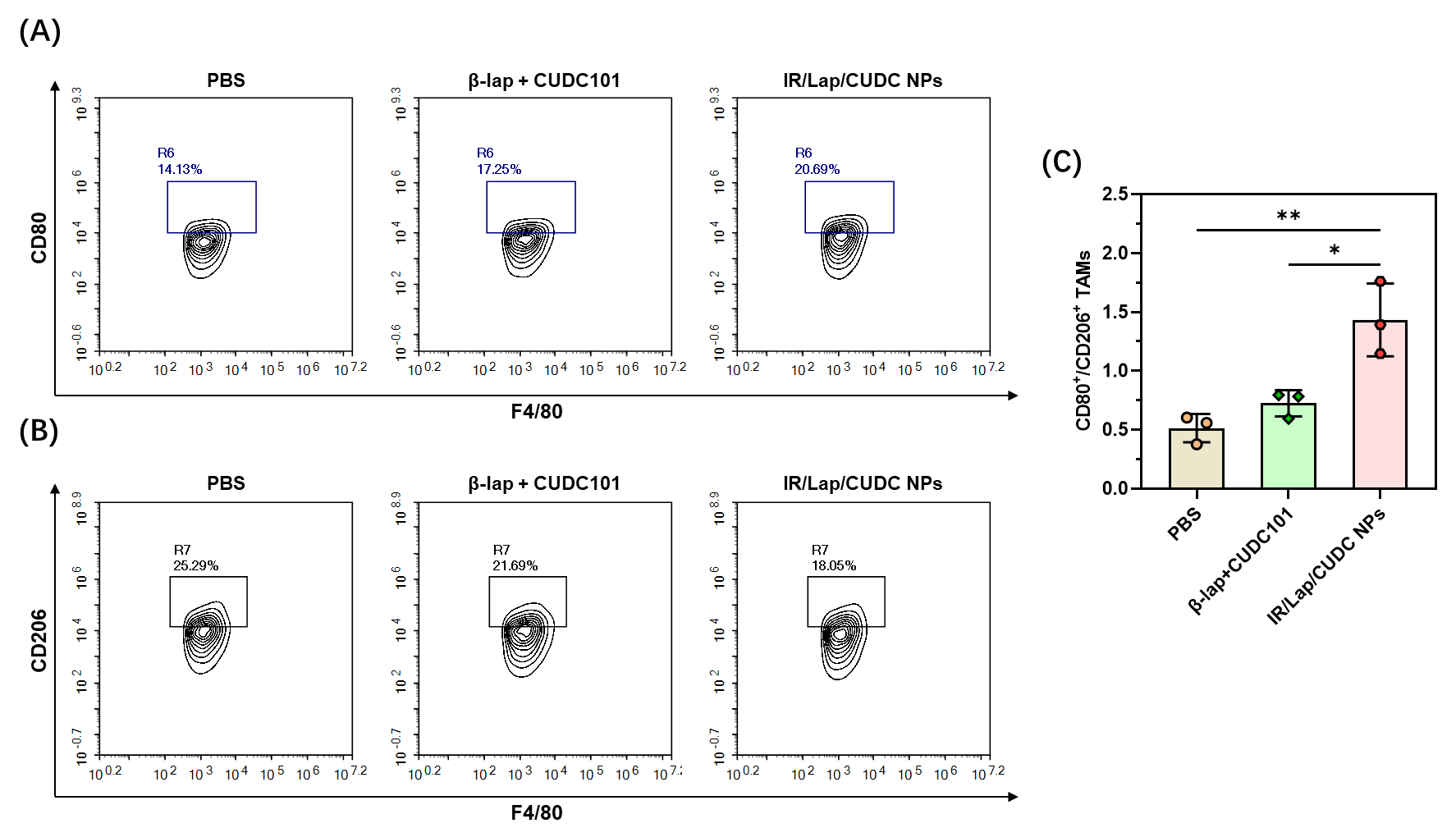


**Figure S23:** (A) Flowcytometric analysis of CD80^+^ anti-tumor macrophages in the primary tumor tissues collected from the treated mice. (B) Flowcytometric analysis of CD206^+^ tumor-promoting macrophages in the primary tumor tissues. (C) Quantitative results of the ratio of CD80^+^ to CD206^+^ tumor-associated macrophages in different groups.


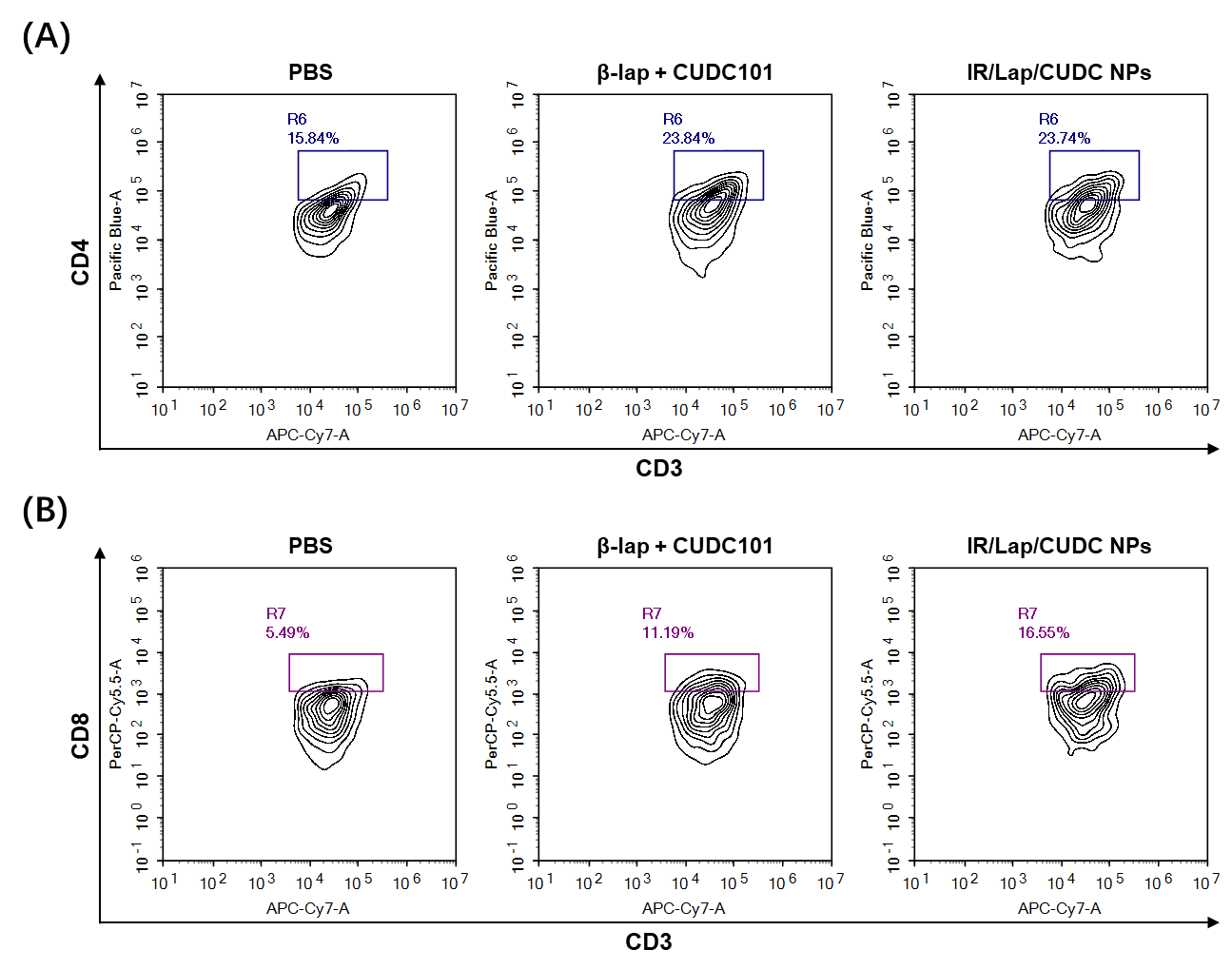


**Figure S24:** (A) Flowcytometric analysis of CD4^+^ T helper cells in the primary tumor tissues collected from the treated mice. (B) Flowcytometric analysis of CD8^+^ cytotoxic T cells in the primary tumor tissues.


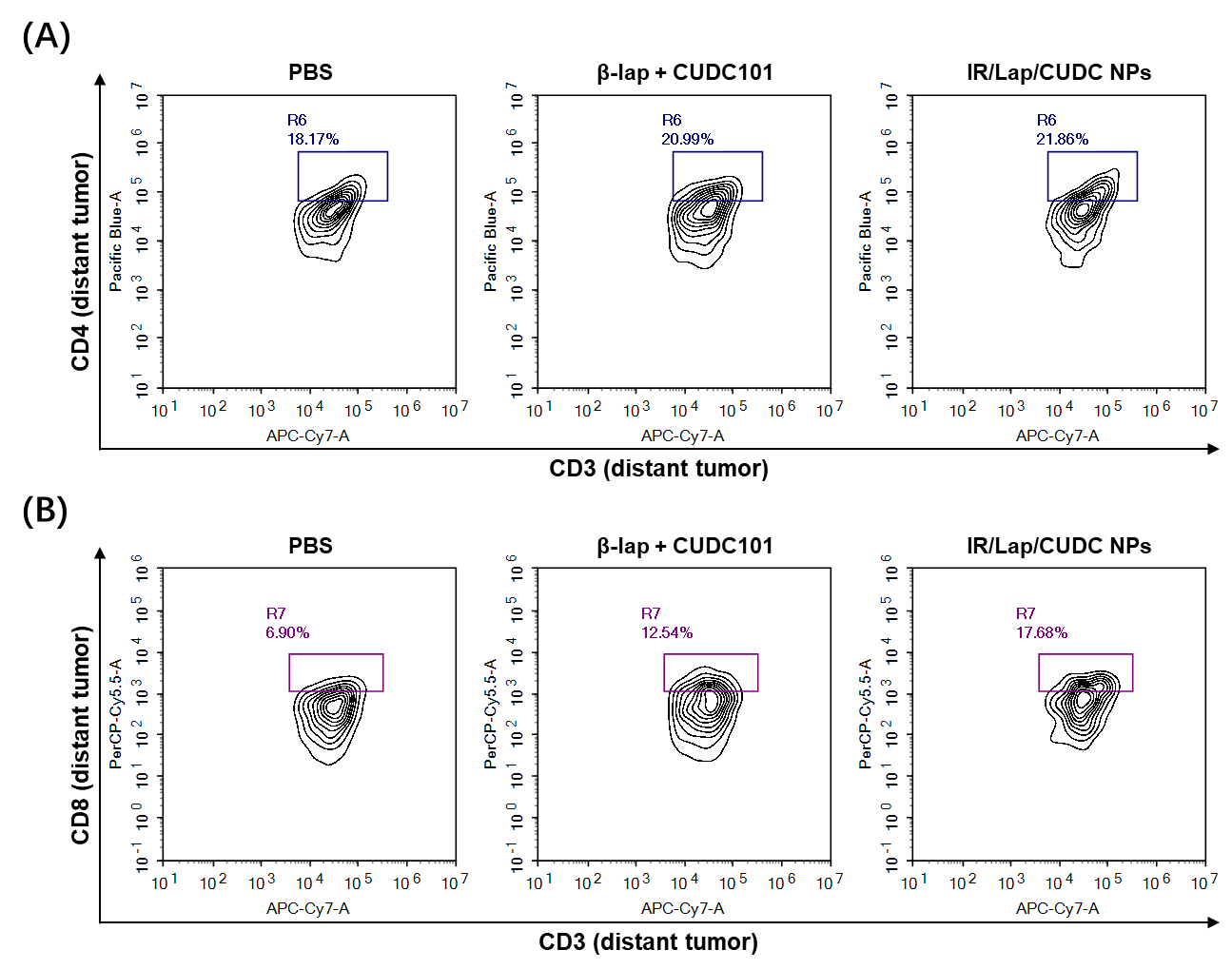


**Figure S25:** (A) Flowcytometric analysis of CD4^+^ T helper cells in the distant tumor tissues collected from the treated mice. (B) Flowcytometric analysis of CD8^+^ cytotoxic T cells in the distant tumor tissues.
